# Construction and Performance Testing of a Fast-Assembly COVID-19 (FALCON) Emergency Ventilator in a Model of Normal and Low-Pulmonary Compliance Conditions

**DOI:** 10.3389/fphys.2021.642353

**Published:** 2021-03-22

**Authors:** Luke A. White, Ryan P. Mackay, Giovanni F. Solitro, Steven A. Conrad, J. Steven Alexander

**Affiliations:** ^1^Department of Molecular and Cellular Physiology, LSU Health Shreveport, Shreveport, LA, United States; ^2^Department of Orthopedic Surgery, LSU Health Shreveport, Shreveport, LA, United States; ^3^Department of Medicine, LSU Health Shreveport, Shreveport, LA, United States; ^4^Department of Emergency Medicine, LSU Health Shreveport, Shreveport, LA, United States; ^5^Department of Pediatrics, LSU Health Shreveport, Shreveport, LA, United States; ^6^Department of Neurology, LSU Health Shreveport, Shreveport, LA, United States

**Keywords:** ventilator, emergency, low-cost, COVID-19, ARDS, severe acute respiratory syndrome

## Abstract

**Introduction:**

The COVID-19 pandemic has revealed an immense, unmet and international need for available ventilators. Both clinical and engineering groups around the globe have responded through the development of “homemade” or do-it-yourself (DIY) ventilators. Several designs have been prototyped, tested, and shared over the internet. However, many open source DIY ventilators require extensive familiarity with microcontroller programming and electronics assembly, which many healthcare providers may lack. In light of this, we designed and bench tested a low-cost, pressure-controlled mechanical ventilator that is “plug and play” by design, where no end-user microcontroller programming is required. This Fast-AssembLy COVID-Nineteen (FALCON) emergency prototype ventilator can be rapidly assembled and could be readily modified and improved upon to potentially provide a ventilatory option when no other is present, especially in low- and middle-income countries.

**Hypothesis:**

We anticipated that a minimal component prototype ventilator could be easily assembled that could reproduce pressure/flow waveforms and tidal volumes similar to a hospital grade ventilator (Engström Carestation^TM^).

**Materials and Methods:**

We benched-tested our prototype ventilator using an artificial test lung under 36 test conditions with varying respiratory rates, peak inspiratory pressures (PIP), positive end expiratory pressures (PEEP), and artificial lung compliances. Pressure and flow waveforms were recorded, and tidal volumes calculated with prototype ventilator performance compared to a hospital-grade ventilator (Engström Carestation^TM^) under identical test conditions.

**Results:**

Pressure and flow waveforms produced by the prototype ventilator were highly similar to the Carestation^TM^. The ventilator generated consistent PIP/PEEP, with tidal volume ranges similar to the Carestation^TM^. The FALCON prototype was tested continuously for a 5-day period without failure or significant changes in delivered PIP/PEEP.

**Conclusion:**

The FALCON prototype ventilator is an inexpensive and easily-assembled “plug and play” emergency ventilator design. The FALCON ventilator is currently a non-certified prototype that, following further appropriate validation and testing, might eventually be used as a life-saving emergency device in extraordinary circumstances when more sophisticated forms of ventilation are unavailable.

## Introduction

Mechanical ventilation is an often lifesaving intervention in intensive care and emergent situations for patients who experience acute respiratory failure, including acute respiratory distress syndrome (ARDS), manifested by progressive hypoxemia, respiratory acidosis, excessive work of breathing, and high respiratory rates (Slutsky, 1993). Commercial ventilators are expensive devices due to numerous features and multiple modes, commonly costing over 25,000 USD. Most hospitals operate with an adequate but limited supply of ventilators as purchase and maintenance costs can be prohibitive (Heather Glass, 2019). A low reserve of ventilators means healthcare capacity could be overwhelmed by a large healthcare crisis. A number of guidelines have been published regarding the management of medical resources, including mechanical ventilators, during emergency and disaster surge situations (Aziz et al., 2020; Frederick and White, 2020; Maves et al., 2020).

Original projections by the Center for Disease Control and Prevention (CDC) estimated that anywhere between 2.4 and 21 million people may ultimately require hospitalization due to COVID-19 (Fink, 2020). Early estimates also suggested that several hundred thousand to greater than 1 million ventilators might be needed to care for COVID-19 patients in the United States alone (Ranney et al., 2020). Based on these realities, the United States Food and Drug Administration (FDA) issued an Emergency Use Authorization (EUA) for guidance on developing new or repurposing non-ICU ventilators to treat COVID-19 (U.S. Food and Drug Administration, 2020a). Support groups around the world rapidly responded by developing low-cost “homemade” mechanical ventilators for use in emergency surge crisis situations such as the COVID-19 pandemic (Albert et al., 2020; Blacker et al., 2020; Galbialta et al., 2020; Garmendia et al., 2020; King et al., 2020; Pearce, 2020; Zuckerberg et al., 2020; MIT Emergency Ventilator, 2020a).

Fortunately, the need for mechanical ventilators in the United States so far have been overestimated; preliminary reporting indicates that only 13.9% of the approximately 130,000 people hospitalized with COVID-19 in 2020 required ventilatory support (Centers for Disease Control and Prevention, 2021). However, satisfactory ventilator supply has posed a greater challenge in rural, low- and middle- income communities (LMICs), where obtaining and maintaining conventional medical-grade mechanical ventilators is not feasible (Guérin and Lévy, 2020). For example, African countries averaged less than 1 ventilator per 100,000 people, at least twenty times lower the rate of the United States, at the start of the pandemic (Houreld et al., 2020). India, a middle-income country, was estimated to have less than 5 ventilators per 100,000 (Soutik, 2020). Furthermore, conventional ventilators that require pressurized gasses to operate cannot be reliably used where access to dependable pressurized gas is unavailable (Fenton, 2020; Madzimbamuto, 2020).

Although low-cost, easy-to-build non-invasive emergency ventilators are simpler in design compared to commercially produced counterparts, they still often require microcontroller programming and electronics assembly (Blacker et al., 2020; Garmendia et al., 2020; MIT Emergency Ventilator, 2020a). While potentially very useful clinically, most healthcare centers cannot assemble and program such devices. In light of these challenges, we designed a low-cost, “plug and play” time-cycled pressure-controlled mechanical ventilator requiring no end-user programming. This simplified design platform can be easily assembled for use by healthcare workers with applications in emergency and disaster surge situations, especially in LMICs, when other preferred ventilatory options are unavailable. We hypothesized that our Fast-AssembLy COVID-Nineteen (FALCON) emergency prototype ventilator could reproduce pressure/flow waveforms and tidal volumes similar to a hospital grade ventilator (Engström Carestation^TM^) in a variety of benchtop tests.

## Materials and Methods

### Ventilator Design

The design of our prototype ventilator was guided by two ideas; namely, (1) a “readily and rapidly assembled” approach, using components that can be easily obtained and utilized by non-engineers, and (2) “plug and play” capability. In computer systems engineering, “plug and play” refers to a feature where peripheral hardware functions immediately as intended once connected without the need for the end-user to manually adjust or reconfigure settings (OED, 2020). Similarly, we use the term “plug and play” to mean that the component parts of the ventilator should work as intended after assembly without the need for the end-user to program or upload any software or code to a microcontroller. For this reason, the use of programmable microcontrollers such as “*Arduino*” or “*Raspberry Pi*” and peripheral input devices necessitating the development and use of specialized coding or programming was deliberately avoided. However, even within such constraints, the prototype ventilator should maintain adequate functionality sufficient for emergency use; in particular, the control and setting of PIP, PEEP, respiratory rate (RR), and inspiratory: expiratory (I:E) ratio.

**FIGURE 1:**
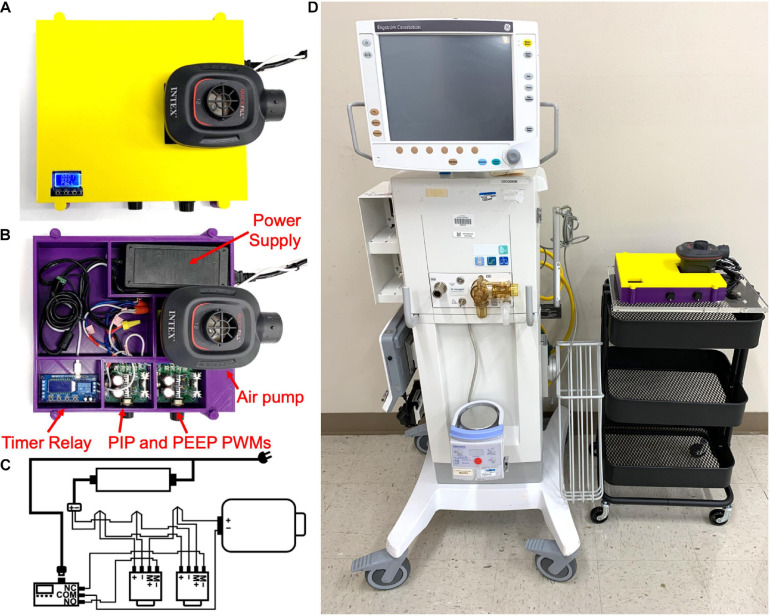
Assembled FALCON prototype ventilator with **(A)** and without **(B)** the housing cover. **(C)** Wiring diagram for the FALCON prototype ventilator. Wires are connected to each other with electrical twist caps and to the 12V power supply, timer relay, and the pulse width modulators (PWMs) controlling peak inspiratory pressure (PIP PWM) and positive end expiratory pressure (PEEP PWM) with screw terminals. A 5V micro USB power adapter is used to power the timer relay (*M*+ = positive motor, *M*- = negative motor, NO = normally open, NC = normally closed, and COM = common). **(D)** Size comparison between the Carestation^TM^ and FALCON prototype ventilator.

**FIGURE 2:**
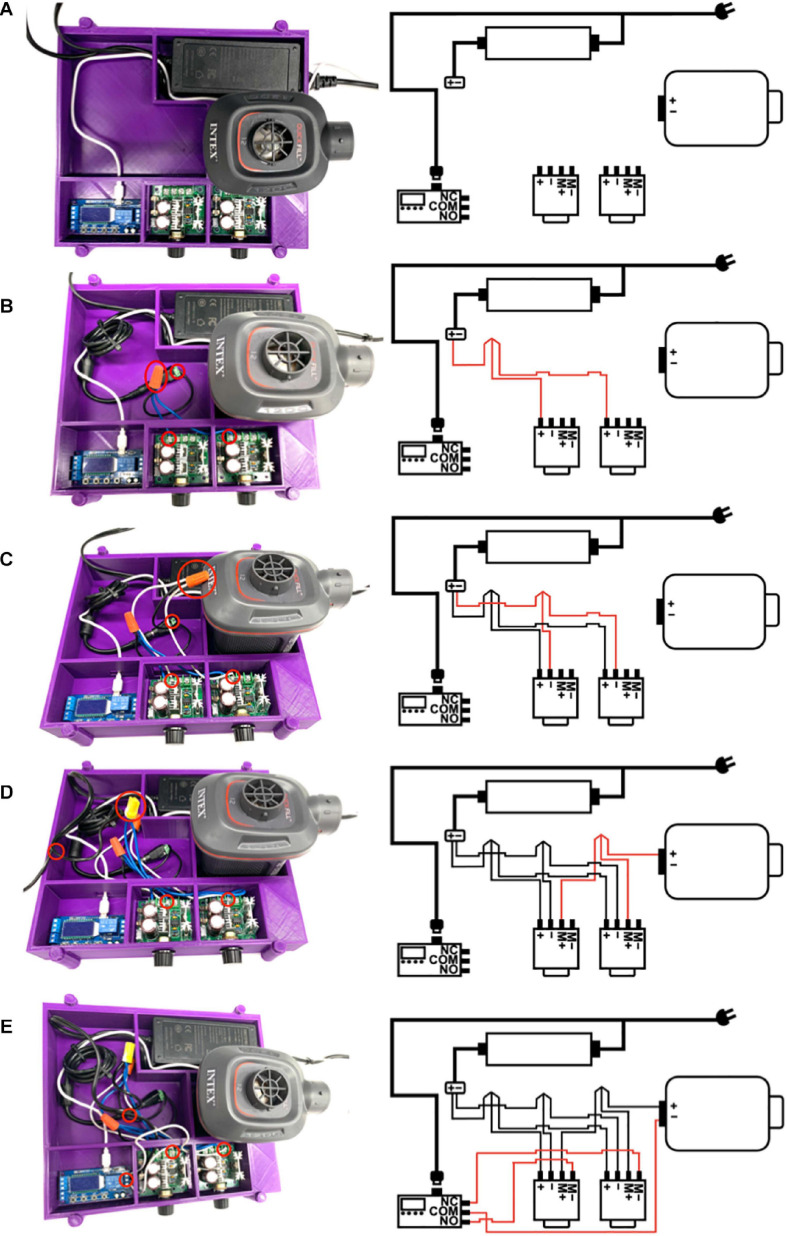
**(A)** The electric air pump, power supply, timer relay, and pulse width modulators (PWMs) are placed inside the 3D printed housing unit. The micro USB 5V power supply is plugged into the timer relay; however, the power supplies are not connected to a power outlet at this time (*M*+ = positive motor, *M*- = negative motor, NO = normally open, NC = normally closed, and COM = common). **(B)** The **+** end of the power supply is connected to the **+** terminals of both PWMs using a screw-on wire cap connector. **(C)** The - end of the power supply is connected to the - terminals of both PWMs using a screw-on wire cap connector. **(D)** The **+** end of the electric air pump is connected to the *M*+ terminals of both PWMs using a screw-on wire cap connector. **(E)** The COM terminal of the timer relay is connected to the – end of the electric air pump, and the NC terminal of the timer relay is connected to the *M*– terminal of the PWM controlling positive end expiratory pressure (PEEP PWM) while the NO terminal of the timer relay is connected to the *M*– terminal of the PWM controlling peak inspiratory pressure (PIP PWM).

Based on this simplified design strategy, we developed and assembled a prototype ventilator (Figure 1A) which was installed in a 3D printed housing (STL printing files available upon request) using computer aided design software (Fusion 360, Autodesk, San Rafael, CA, United States) and 3D printed (TAZ Workhorse; Lulzbot, Fargo, ND, United States) using polylactic acid filament (2.85 mm PLA+; eSUN^®^, Shenzhen, China). All the necessary parts used to assemble the ventilator are shown in Figure 1B, with the exception of a pressure gauge, and a list of purchased parts for assembling the prototype ventilator is found in the Supplementary Material. Respiratory rate is set by a timer relay (XY-LJ02, Belong International Co., Shanghai, China). A 12-volt 8-amp electric air pump (Quick-Fill DC Electric Air Pump AP636, Intex, Long Beach, CA, United States; maximal air flow 650 L/min) provides air flow and pressure delivered to the recipient. The timer relay continuously alternates between “ON” and “OFF” states, controlling the air pump at times set on the programmable relay. The operator sets the inspiratory and expiratory times by setting the durations of the “ON” and “OFF” states, respectively. The air pump is controlled by two pulse width modulators (PWMs; 12-40V 8A PWM DC, Riorand^TM^, Richmond, Canada) one PWM for PIP (“ON”) pressure state and a second PWM control for the PEEP (“OFF”) state. A pressure gauge (NS 60 PBS; Instrumentation Industries Inc, Bethel Park, PA, United States), is used to confirm pressures and adjust the PIP and PEEP values by effectively altering the current delivered to the air pump during “ON” and “OFF” states, respectively. The timer relay is powered by a 5-volt 1-amp micro USB power adapter (PT-WC-05, UorMe, Shanghai, China) and micro USB to USB cable (HST-SMT3001, SmallElectric, Dongguan, China). With the exception of the pressure gauge, all parts together were purchased for less than 75 USD. Importantly, our design requires no soldered connections, only 13 screw terminals, and 3 wire cap connections; the wiring diagram is shown in Figure 1C. A size comparison of the prototype ventilator to a hospital-grade mechanical ventilator (Engström Carestation^TM^, General Electric Healthcare, Chicago, IL, United States) is shown in Figure 1D. Step-by-step assembly instructions for the FALCON ventilator are shown in Figure 2 (see Supplementary Material for detailed assembly instructions). Settings for timer circuit and adjusting the I:E ratio are also provided in the Supplementary Material.

### Model Benchtop Testing

#### Ventilator Output Airflow and Pressure Monitoring

**FIGURE 3:**
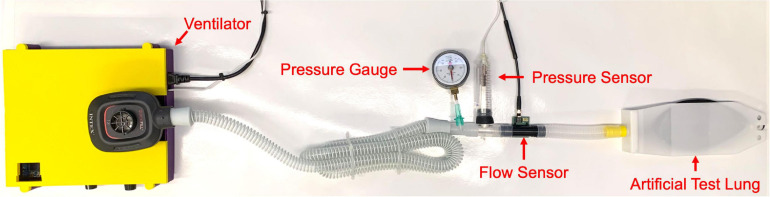
Experimental setup for benchtop testing.

We created a flow and pressure monitoring system for testing the FALCON prototype ventilator (Figure 3) and comparing functionality to a commercially available hospital ventilator. A test lung (Siemens Maquet Adult 190 Test Lung, Getinge, Göteborg, Sweden) was connected to the FALCON ventilator using 22 mm silicone rubber fitted ribbed pressure hosing (6 ft. × 19 mm inner diameter, Philips Respironics, Murrysville, PA, United States) and standard plastic ventilator tubing (0.7 mm thickness, 22 mm inner diameter). Rates of air flow were evaluated using a calibrated differential pressure sensor (Evaluation Kit EK-P4, Sensirion AG, Staefa, Switzerland) connected in-line between the ventilator and the test lung. A highly sensitive air pressure sensor (DPS310, Infineon Technologies AG, Neubiberg, Germany) was also placed between the ventilator and test lung to measure pressure profiles. A pressure gauge was also oriented in-line so that PIP and PEEP could be monitored by the operator and adjusted. These sensors captured flow and pressure data at rates of ∼200 and 25 Hz, respectively; these values were transferred to a computer (Optiplex 7070, Dell Inc., Round Rock, TX, United States) for later analysis.

#### Benchtop Testing

Thirty-six test scenarios were compared using the FALCON prototype ventilator (Table 1) and Carestation^TM^ ventilator. The Carestation^TM^ was set in mandatory pressure-controlled ventilation for comparison testing with the FALCON prototype (FIO_2_ = 0.21, inspiratory rise time = 100 ms, further parameters for each test condition specified in Table 1). The test lung was evaluated at two different levels of pulmonary compliance (“normal” = 74 mL/cm H_2_O and “restrictive” = 34 mL/cm H_2_O), over a range of respiratory rates and target PIP and PEEP values. Airway resistance was fixed at 12 cm H_2_O⋅s/L. At the beginning of each test for the prototype ventilator, the inspiratory and expiratory pressures were adjusted with the “PIP” and “PEEP” PWMs by the operator until the pressure gauge read the appropriate target PIP and PEEP.

**TABLE 1:** Conditions used in benchtop testing (RR = respiratory rate, in breaths per min; PIP = peak inspiratory pressure, in cm H_2_O; PEEP = positive end expiratory pressure, in cm H_2_O; normal lung compliance = 74 mL/cm H_2_O; restrictive lung compliance = 34 mL/cm H_2_O).

**Condition**	**Compliance**	**RR**	**PIP**	**PEEP**	**Condition**	**Compliance**	**RR**	**PIP**	**PEEP**
1	Normal	10	10	0	19	Restrictive	10	10	0
2	Normal	10	10	5	20	Restrictive	10	10	5
3	Normal	10	15	5	21	Restrictive	10	15	5
4	Normal	10	15	10	22	Restrictive	10	15	10
5	Normal	10	20	10	23	Restrictive	10	20	10
6	Normal	10	20	15	24	Restrictive	10	20	15
7	Normal	20	10	0	25	Restrictive	20	10	0
8	Normal	20	10	5	26	Restrictive	20	10	5
9	Normal	20	15	5	27	Restrictive	20	15	5
10	Normal	20	15	10	28	Restrictive	20	15	10
11	Normal	20	20	10	29	Restrictive	20	20	10
12	Normal	20	20	15	30	Restrictive	20	20	15
13	Normal	30	10	0	31	Restrictive	30	10	0
14	Normal	30	10	5	32	Restrictive	30	10	5
15	Normal	30	15	5	33	Restrictive	30	15	5
16	Normal	30	15	10	34	Restrictive	30	15	10
17	Normal	30	20	10	35	Restrictive	30	20	10
18	Normal	30	20	15	36	Restrictive	30	20	15

Benchtop tests using identical test conditions for the prototype and Carestation^TM^ ventilators were run on the same day. At the beginning of each test session, zero-pressure measurements were acquired. Each test condition was run for at least 10 respiratory cycles. The PIP and PEEP were determined for each cycle. Tidal volumes were computed for each cycle by calculating the area under the flow curve during inspiration, and the average tidal volume from each benchtop test was calculated. The set of average tidal volumes produced by the prototype FALCON ventilator was compared to the set of average tidal volumes produced by the Carestation^TM^ ventilator using a two-tailed, paired Students *t*-test. A *p* value < 0.05 was considered statistically significant. Statistics were performed with the Graphpad Prism statistics software (version 8.4, Graphpad, San Diego, CA, United States).

#### Duration Benchtop Testing

In a separate test, the FALCON prototype ventilator was run continuously for a 5-day period (PIP = 15 cm H_2_O, PEEP = 5 cm H_2_O, RR = 10 breaths per min, and normal lung compliance). PIP, PEEP, and RR were set at the start of the test and not adjusted further for the remainder of the 5-day testing period. Pressures were recorded daily for 9 to 10 consecutive respiratory cycles. The ventilator was briefly disconnected from the test circuit each day after pressure measurements in order to acquire zero-pressure measurements.

## Results

### The FALCON Prototype Ventilator Recapitulates Pressure and Flow Waveforms Produced by a Conventional Hospital Ventilator

**FIGURE 4:**
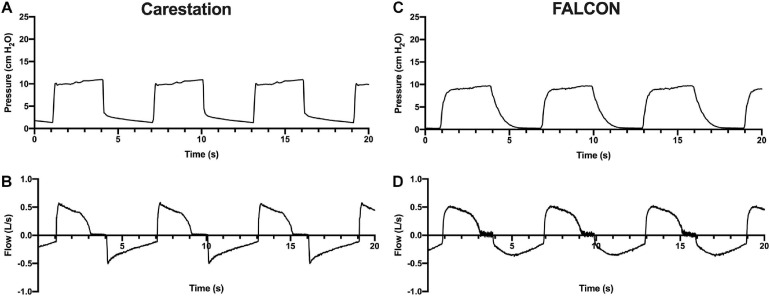
Pressure (in cm H_2_O) and flow (in L/s) curves generated by the Carestation^TM^ **(A,B)** and FALCON prototype **(C,D)** ventilators over a 20 s period during test condition 1 (peak inspiratory pressure = 10 cm H_2_O, positive end expiratory pressure = 0 cm H_2_O, respiratory rate = 10 breaths per min, and normal lung compliance of 74 mL/cm H_2_O). The remaining pressure and flow curves can be seen in Supplementary Figure 1.

The pressure and flow waveforms generated during the ten consecutive respiratory cycles were highly uniform during each benchtop test for both the FALCON prototype and Carestation^TM^ (Figure 4 shows representative waveforms and Supplementary Figure 1 shows waveforms generated during all 36 test conditions). Pressure profiles for the FALCON ventilator were less abrupt and changed more gradually between inspiration and expiration compared to the sharper square-like waveforms produced by the Carestation^TM^ (Figures 4A,C). Likewise, flow waveforms from the prototype ventilator also peaked less sharply and developed slightly more gradually than flow waveforms generated by the Carestation^TM^ (Figures 4B,D).

### The FALCON Prototype Ventilator Consistently Generated Peak Inspiratory and Positive End Expiratory Pressures Close to Target Values

**FIGURE 5:**
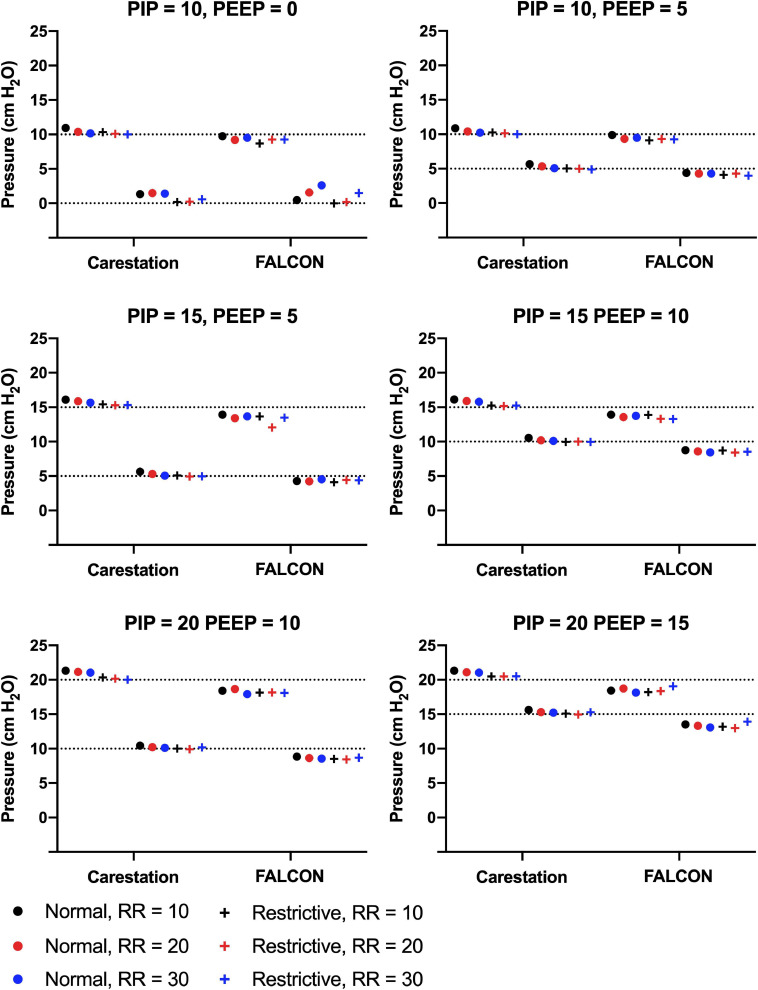
Mean peak inspiratory pressure (PIP) and positive end expiratory pressure (PEEP) generated by the Carestation^TM^ and FALCON prototype ventilators measured from 10 consecutive respiratory cycles in all test conditions. Data are grouped by target PIP and PEEP with varying lung compliance (normal = 74 mL/cm H_2_O or restrictive = 34 mL/cm H_2_O) and respiratory rate (RR; 10, 20, or 30 breaths per min). Dotted lines indicate target PIP and PEEP.

The mean PIP and PEEP were calculated from ten consecutive respiratory cycles (Figure 5). The mean standard deviation of PIP and PEEP from consecutive replicate readings over the course of a test for all benchtop tests were 0.03 cm H_2_O and 0.07 cm H_2_O, respectively, for the Carestation^TM^ and 0.05 cm H_2_O and 0.07 cm H_2_O for the FALCON prototype. The PIPs generated by the Carestation^TM^ were greater than the target PIPs (mean pressure difference to target = 0.6 cm H_2_O, standard deviation = 0.4 cm H_2_O, maximal pressure difference to target = 1.3 cm H_2_O in condition 5). On average, the PEEPs produced by the Carestation^TM^ were also greater than the target PEEP (mean pressure difference to target = 0.3 cm H_2_O, standard deviation = 0.4 cm H_2_O, max pressure difference = 1.5 cm H_2_O in condition 7). In contrast, the average PIP (mean pressure difference to target = −1.3 cm H_2_O, standard deviation = 0.6 cm H_2_O, and max pressure difference = −2.9 cm H_2_O in condition 27) and PEEP (mean difference to target = −0.8 cm H_2_O, standard deviation = 1.0 cm H_2_O, and max pressure difference = 2.6 cm H_2_O in condition 13) produced by the FALCON prototype ventilator were slightly lower than the target pressures.

### Tidal Volumes Produced by the FALCON Prototype Ventilator Are Similar to a Conventional Hospital Ventilator

**FIGURE 6:**
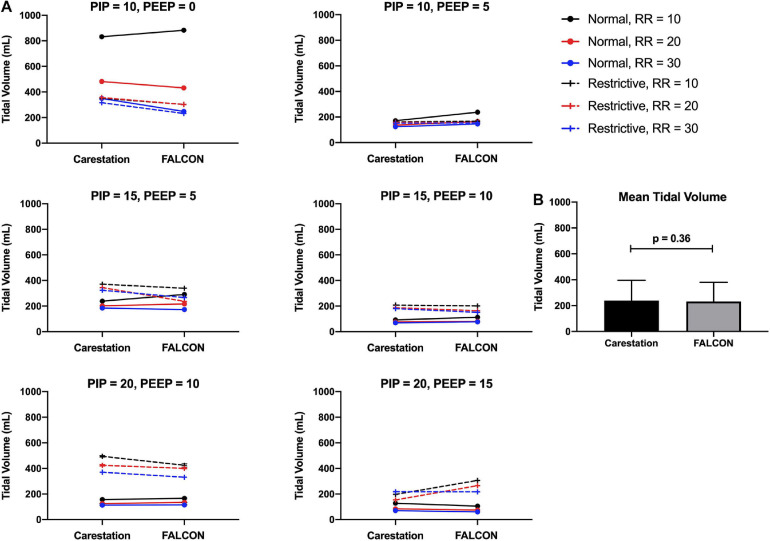
**(A)** Mean tidal volumes produced by the Carestation^TM^ and FALCON prototype ventilators calculated from 10 consecutive respiratory cycles. Data are paired with identical test conditions for the Carestation^TM^ and prototype ventilators and grouped by target peak inspiratory pressure (PIP) and positive end expiratory pressure (PEEP) with varying lung compliance (normal = 74 mL/cm H_2_O or restrictive = 34 mL/cm H_2_O) and respiratory rate (RR; 10, 20, or 30 breaths per min). **(B)** Mean tidal volumes generated by the Carestation^TM^ and prototype ventilators under all 36 test conditions (*p* = 0.36, two-tailed paired Students *t*-test, error bars indicate standard deviation).

Tidal volumes delivered by the Carestation^TM^ and prototype ventilators were determined by calculating the average area under the flow curve during the inspiratory phase from ten consecutive respiratory cycles. The mean standard deviation of tidal volume from consecutive replicate readings over the course of a test for all benchtop tests were 3 mL for both the Carestation^TM^ and FALCON prototype ventilators. Since both ventilators were pressure-controlled, there was no “target” tidal volume for a given set of test conditions. Instead, the tidal volumes delivered by the Carestation^TM^ and prototype ventilators were paired based on test condition and compared (Figure 6A). A paired two-tailed *t*-test of the tidal volumes grouped by condition found no statistically significant difference (*p* = 0.36, Figure 6B) between the tidal volumes delivered by the Carestation^TM^ (mean = 239 mL, standard deviation = 156 mL) versus the FALCON prototype ventilator (mean = 232 mL, standard deviation = 149 mL).

### The FALCON Prototype Ventilator Reliably Generates Target Pressures Over an Extended Period of Time

**FIGURE 7:**
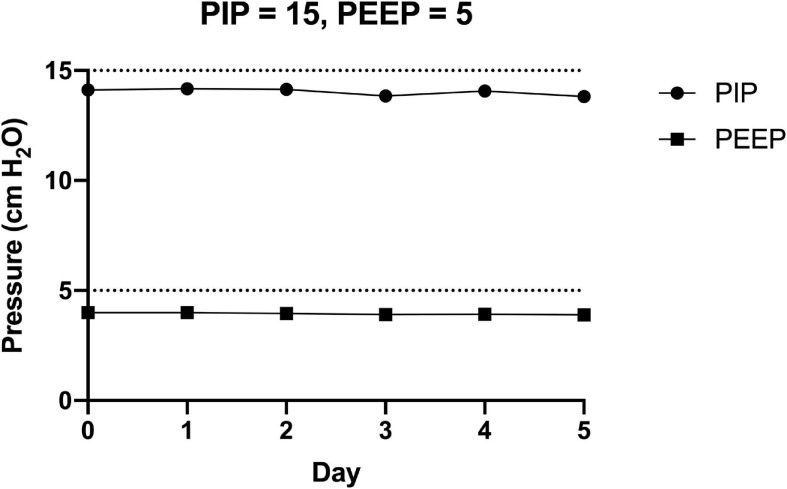
The FALCON prototype ventilator was connected to the experimental setup and run (peak inspiratory pressure = 10 cm H_2_O, positive end expiratory pressure = 5 cm H_2_O, respiratory rate = 10 breaths per min, and normal lung compliance of 74 mL/cm H_2_O) continuously for 5 days. Pressures were recorded for 10 consecutive respiratory cycles each day, and the mean peak inspiratory pressure (PIP) and positive end expiratory pressure (PEEP) for each day are shown.

The FALCON prototype ventilator was run continuously for 5 days (PIP = 15 cm H_2_O, PEEP = 5 cm H_2_O, RR = 10 breaths per min, and normal lung compliance). The mean PIP and PEEP from ten consecutive respiratory cycles were measured daily (Figure 7). On Day 0, the prototype ventilator produced pressures slightly less than the target pressures (PIP = 14.1 cm H_2_O, PEEP = 4.0 cm H_2_O). By Day 5, the pressures decreased by 2 percent (PIP = 13.8 cm H_2_O, PEEP = 3.9 cm H_2_O), and the day-to-day standard deviations of the mean PIP and PEEP were 0.2 cm H_2_O and <0.1 cm H_2_O, respectively.

## Discussion

The FALCON ventilator is a stable disaster/emergency “plug and play” ventilator that can be readily and rapidly assembled from widely available and inexpensive components. Several other groups have also developed mechanical ventilators, and many of these designs have been shared on the web as “open-source” platforms with several diverse features (Frazer et al., 2020). Several of the proposed designs have not yet been assembled; fewer have been tested for utility, and end users may have to fill in gaps of assembly and programming with unspecified hardware and software (Pearce, 2020).

Many of these ventilator designs generate pressure and flow through mechanical compression of a self-inflating bag valve mask, or BVM (Blacker et al., 2020; MIT Emergency Ventilator, 2020a). Bag ventilation relies on adjustment of manual compression to provide volume-controlled ventilation. Many bag-based ventilators contain mechanical moving parts that can operationally fail during the lifetime of the ventilator (MIT Emergency Ventilator, 2020b). Another recent and inexpensive (∼10 USD) “minimalist” prototype ventilator by Chi et al. is 3D printed, and it relies on pressurized gas either from an air tank or hospital wall supply to accomplish ventilation (Albert et al., 2020). While inexpensive, reliance on pressurized gas may also be impractical during emergency surge crises, especially in LMICs when pressurized gas may not be readily accessible or sufficiently pressurized (Fenton, 2020; Madzimbamuto, 2020). Other prototype ventilators use low-cost electric fans or blowers to produce pressure-controlled ventilation. Garmendia et al. (2020) designed and tested this type of low-cost, fan-powered ventilator that could even be used in non-invasive ventilation scenarios. However, the use of complex, microcontroller programming and peripheral input/output devices remain significant barriers to assembly and use by end-users. Even when all files and instructions are made available to the end-user, assembly, initialization, and calibration may be required for operation.

Therefore, we designed the FALCON as a basic pressure-controlled ventilator that can be assembled without the use of sophisticated microcontrollers. PIP and PEEP values measured over a 5-day testing period were highly consistent, demonstrating minimal pressure “drift” over time. Such consistent pressure delivery are essential; variations in pressures could lead to ventilator induced lung injury if pressures are too high or hypoxemia and respiratory acidosis if pressures are too low. Furthermore, ventilator failure is of particular concern with “homemade” ventilators. For example, bag valve mask ventilators are at risk of mechanical failure due to the use of several moving parts (Nacharaju et al., 2020). The lack of either mechanical or electrical failures during the 5-day testing period supports the robustness of the FALCON prototype ventilator. Nevertheless, the proper functioning of the FALCON prototype ventilator is critically dependent upon the performance of the selected electric air pump and its internal turbine.

Both inspiratory and expiratory pressures in the FALCON prototype are generated by an electric turbine. This approach produces more rounded pressure and flow curves compared to the Carestation^TM^, reflecting rotational inertia of the air pump’s internal fan, as the fan takes a fraction of the expiratory time to passively slow down and stop. At high enough respiratory rates with low target PEEP, there may be insufficient time for the turbine to decelerate, leading to above-target PEEP. This was observed in the progression of increased PEEP from test condition 1 (RR = 10 breaths per min, measured PEEP = 0.5 cm H_2_O), to test condition 7 (RR = 20 breaths per min, measured PEEP = 1.5 cm H_2_O), and finally to test condition 13 (RR = 30 breaths per min, measured PEEP = 2.6 cm H_2_O).

Several benchtop test conditions were intentionally set outside of clinically relevant parameters; these flow curves and calculated tidal volumes are reported for only demonstration purposes. For example, in test condition 10, the PIP and PEEP were set to 20 and 15 cm H_2_O, respectively, but were used in a lung model that was considered “compliant.” This pattern led to an over-inflated lung during expiration with low tidal volumes and was seen in both the prototype FALCON (76 mL) and Carestation^TM^ (81 mL) ventilators; however, this could be within normal parameters for a lung with less than normal compliance.

The testing ranges in this report only cover respiratory mechanics of a subset of patients with COVID-19 induced ARDS (Fan et al., 2020; Haudebourg et al., 2020; Ziehr et al., 2020). Our current study was limited to a lung model with airway resistance values fixed at 12 cm H_2_O⋅s/L and compliances of 74 and 34 mL/cm H_2_O which are relevant to mild to moderate, but not severe, ARDS. We acknowledge that our prototype would therefore not be appropriate for patients with severe ARDS whose lung compliance could be lower, and airway resistance higher, than the tested values. These patients would be ideally treated with a commercial ICU ventilator, where the FALCON prototype would represent an intermediate treatment approach which could make more sophisticated and expensive ICU ventilators available. Future *in vivo* studies testing the efficacy of the prototype ventilator in an animal model of ARDS could be useful in determining the effectiveness of the FALCON prototype ventilator in a wider range of ARDS disease presentation.

The FALCON prototype ventilator is a continuous mandatory ventilator (CMV), where breaths are delivered based on set variables without attempting to sense or respond to patient breathing efforts. Mandatory ventilation has some important disadvantages over assisted/supported ventilation. These can include greater use of sedatives/neuromuscular blockade as well as diaphragmatic dysfunction. Both factors can prolong the need for mechanical ventilation and carry risk for apnea upon ventilator failure (Kress et al., 2000; Sassoon et al., 2004; Gilstrap and MacIntyre, 2013) which anticipates the need for more continuous patient monitoring. In our model, we explored the addition of controls for patient synchrony but realized this would substantially increase the complexity of the FALCON ventilator and reduce its “plug and play” utility. Therefore, it is important to restate that the FALCON ventilator is not intended to replace ICU ventilators for prolonged ventilation but was designed chiefly as a low-cost temporary ventilatory option in surge crises until patients can be transitioned to more conventional, longer-term medical/ICU ventilators.

Cleaning and disinfection of ventilators after use are important steps to minimize exposure to personnel and patients that are subsequently connected to the ventilator (World Health Organization, 2014; Rutala et al., 2019). Depending on the ventilatory circuit set up, the FALCON prototype ventilator should be at similar risk to contamination as more conventional hospital-grade ventilators and should undergo similar cleaning and disinfection protocols (World Health Organization, 2014). In particular, the use of a three-way, two-position pneumatically driven valve, such as those commonly found in BVMs between the bag outlet and mask, could be used to set up inspiratory (from ventilator to valve) and expiratory (from valve to atmosphere) lines with a common line from the valve to the patient. This setup should prevent most respiratory secretions from entering the inspiratory line and contaminating the electric air pump. Additionally, respiratory HEPA filters can be placed along the inspiratory and expiratory lines to eliminate significant viral particle aerosolization (Ari et al., 2016; Ari, 2020; Directorate of Health Services, 2020; Fink et al., 2020). After use, this ventilator can be unplugged and housing surfaces cleaned with conventional solutions used for highly infectious materials, such as 0.26% sodium hypochlorite (Directorate of Health Services, 2020). Ventilator tubing may be reused if it is thoroughly washed with detergent, rinsed, and then subjected to a disinfection solution, such as 0.1% sodium hypochlorite, ensuring that the tubing is flushed adequately (World Health Organization, 2014). Due to the “plug and play” nature of the ventilator, if an electrical component does inadvertently become contaminated, it can be replaced relatively inexpensively.

In its current state of development, although the FALCON ventilator does not have a built-in over-pressure safety valve, the maximal pressure measured from the air pump (when the PWM is set to its maximal value and the outlet is completely occluded) is 36 cm H_2_O (*data not shown*). This pressure is similar to the pressure at which common BVM relief valves are set to open (Ambu^®^ Spur^®^ Ii Datasheet, 2021; Laerdal, 2021). Nevertheless, a relief valve could be placed in-line between the ventilator and patient to prevent overpressure and minimize the risk of pneumothorax.

In order to preserve the “plug and play” characteristic of the prototype ventilator, the need to use standalone, modular monitors and devices with built-in alarms to alert the user of events such as a disconnection or insufficient tidal volume delivery—which are not widely used and may not be readily available—would be necessary to meet FDA recommended safety regulations (Corey et al., 2020; U.S. Food and Drug Administration, 2020b). Other limitations of the FALCON prototype ventilator include lack of control over the fraction of inspired oxygen (FiO_2_) above room air and the lack of internal device checks. Further studies are needed to confirm the biocompatibility of material and the effects of various sterilization techniques and cleaners on the device. Additional studies could also be performed to determine the effects of electromagnetic interference on the device, the production of electromagnetic emissions by the device, the testing of vaporization of lubricants and the outgassing of plastics from the air pump, and the effects on temperature and humidity variation on device performance.

The FALCON prototype device was not constructed or tested under International Organization for Standardization’s (ISO) standards for medical devices (ISO 13485:2016) and is not certified by the FDA’s EUA or other regulatory agency for human use (U.S. Food and Drug Administration, 2020a). The FALCON ventilator is a non-certified device that might eventually be used as a desperate life-saving strategy in extraordinary circumstances when other ventilation choices are unavailable (a table containing the most likely failures, their causes and effects is provided in the Supplementary Material). We recognize that use of more sophisticated commercial ventilators with extensive safety features, self-checks, and functionalities that meet and exceed regulatory standards should always be used when available, and do not advocate substitution of emergency ventilatory strategies on patients as the one described herein.

In conclusion, we have described and benchtop tested the FALCON prototype ventilator as an inexpensive, versatile and readily-assembled emergency “plug and play” mechanical ventilator which demonstrated delivery of pressure and flow waveforms and tidal volumes comparable to a commercial available ventilator (Carestation^TM^). Additionally, this prototype was able to deliver consistent and continuous ventilation over a 5-day period.

## Data Availability Statement

The raw data supporting the conclusions of this article will be made available by the authors, without undue reservation.

## Author Contributions

LW, GS, SC, and JA contributed to conception and design of the study. LW and RM collected and analyzed data. LW wrote the first draft of the manuscript. LW, RM, and JA wrote sections of the manuscript. All authors contributed to manuscript revision, read, and approved the submitted version.

## Conflict of Interest

The authors declare that the research was conducted in the absence of any commercial or financial relationships that could be construed as a potential conflict of interest.
