# Supplementary material for: Construction and Performance Testing of a Fast-Assembly COVID-19 (FALCON) Emergency Ventilator in a Model of Normal and Low-Pulmonary Compliance Conditions
Source: Front Physiol. 2021 Mar 22;12:642353. doi: 10.3389/fphys.2021.642353 (PMC8044930; doi:10.3389/fphys.2021.642353)
Supplement: Supplementary file 1 [file Data_Sheet_1.PDF]

Supplementary Figure 1. Pressure (in cm H2O) and flow (in L/s) curves generated by the Carestation™ and prototype ventilators in a twenty second period during all 36 test conditions.

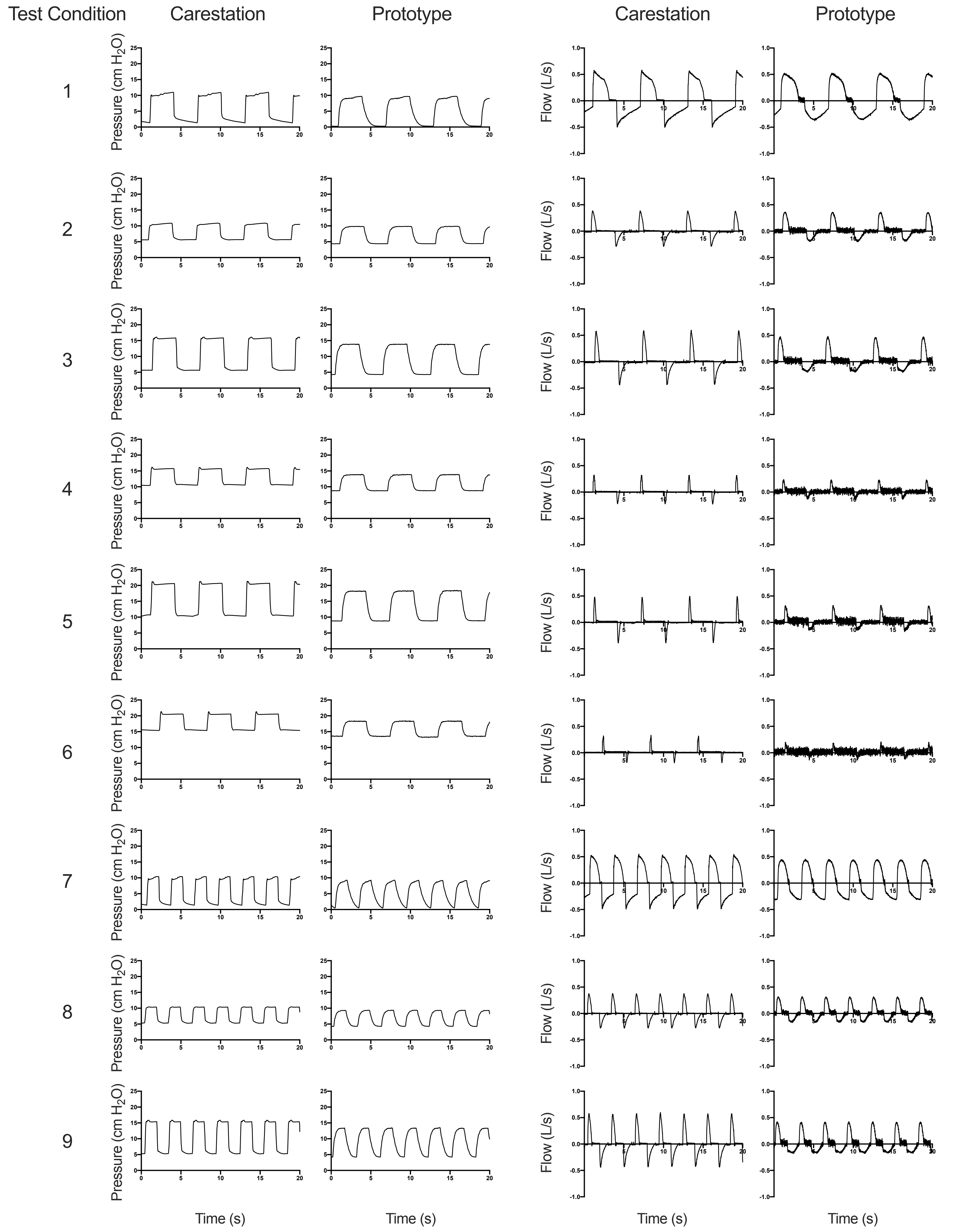

Test Condition
Carestation
Prototype
Carestation
Prototype
1
2
3
4
5
6
7
8
9
Pressure (cm H O)
Flow (L/s)
Time (s)

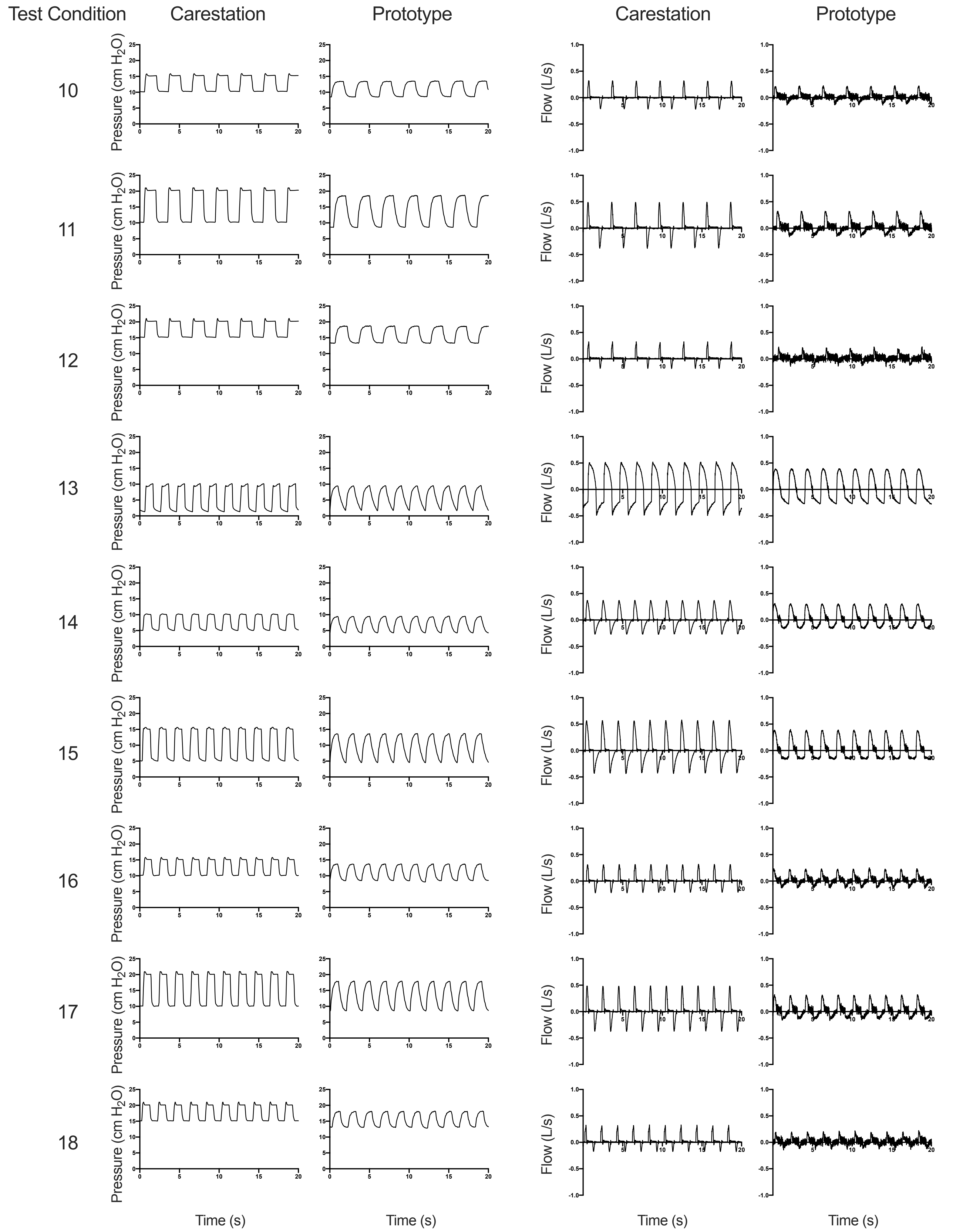
Test Condition
Carestation
Prototype
Carestation
Prototype
10
11
12
13
14
15
16
17
18
Pressure (cm H2O)
Flow (L/s)
Time (s)

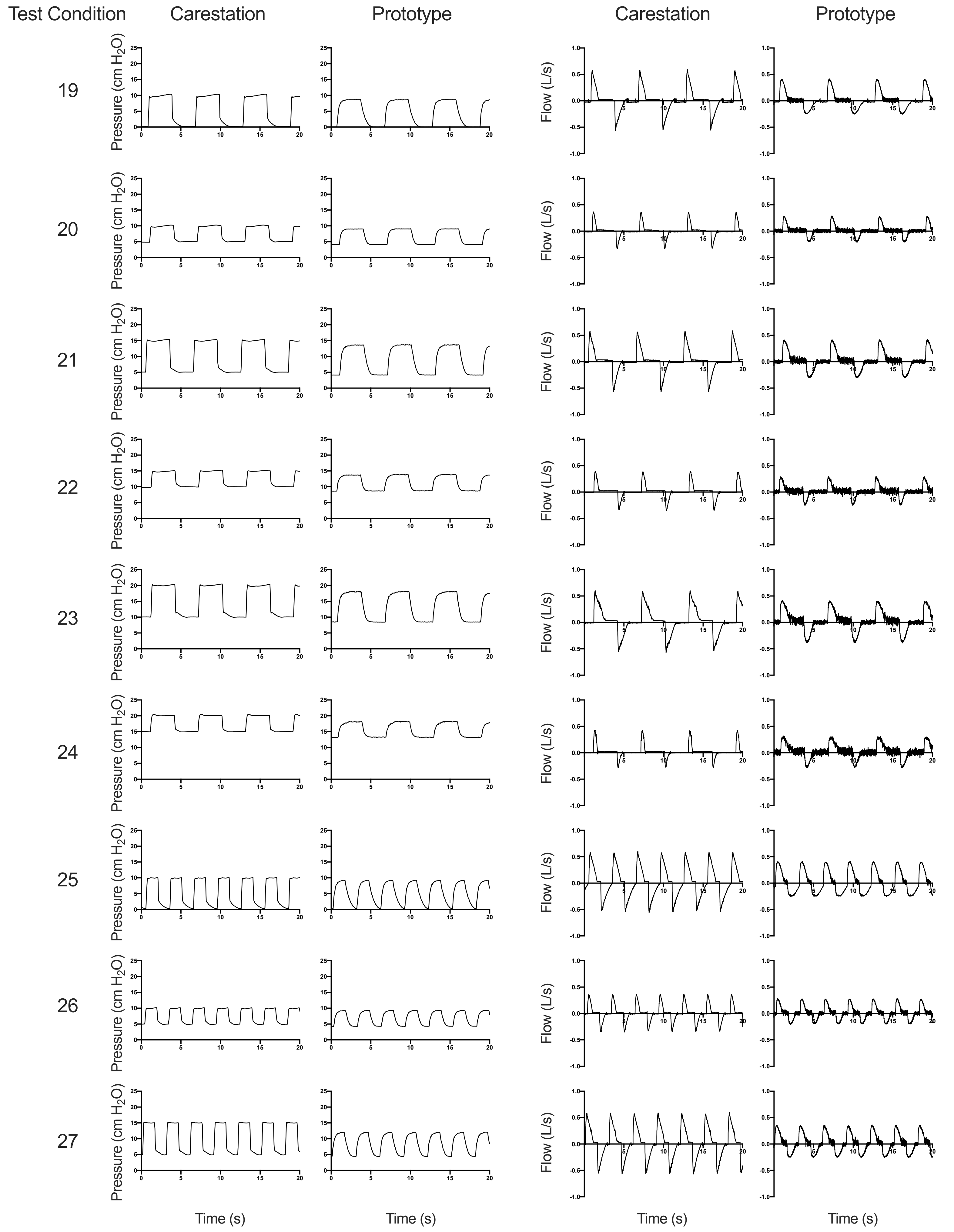
Test Condition
Carestation
Prototype
Carestation
Prototype
19
20
21
22
23
24
25
26
27
Pressure (cm H2O)
Flow (L/s)
Time (s)

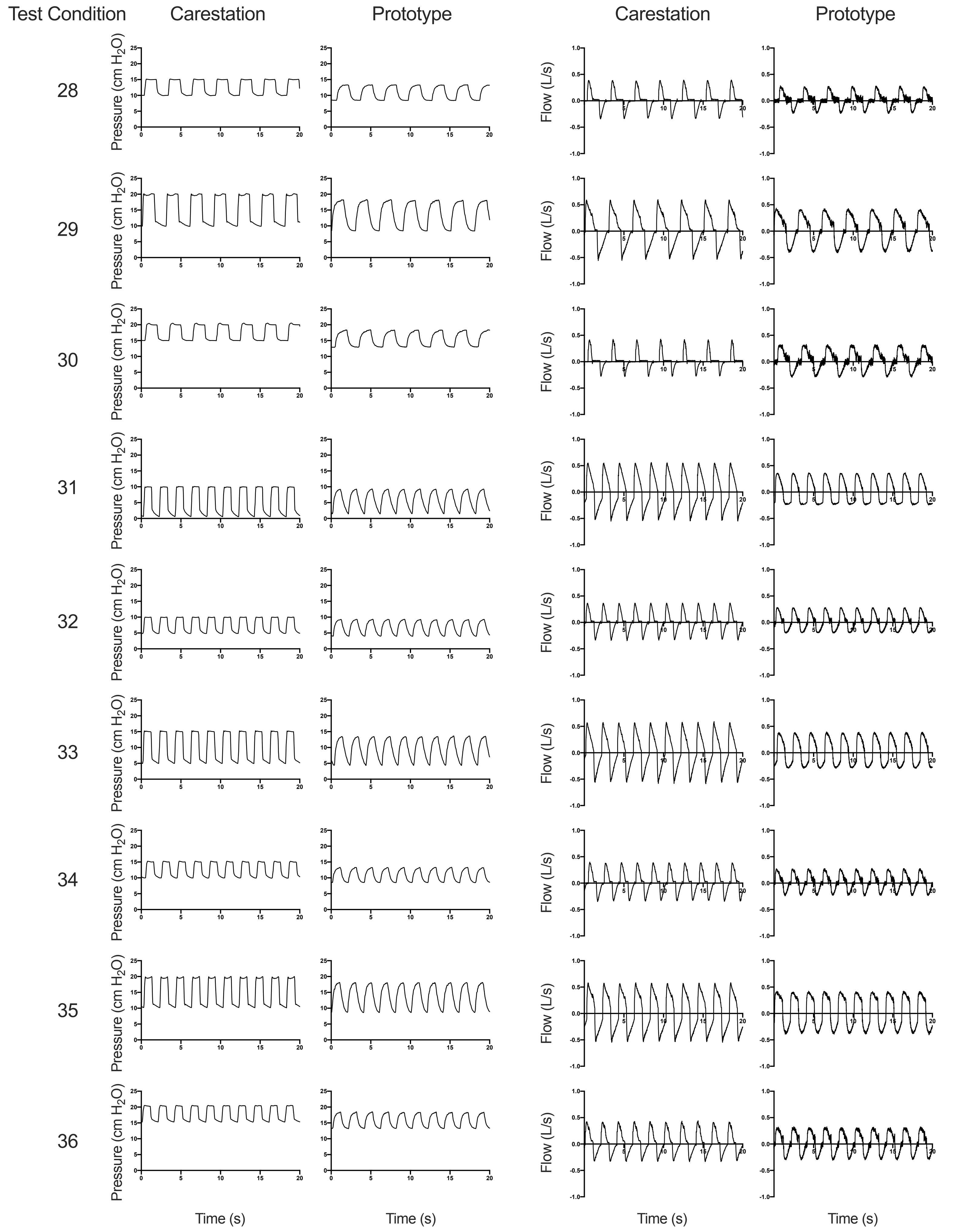
Test Condition
Carestation
Prototype
Carestation
Prototype
28
29
30
31
32
33
34
35
36
Pressure (cm H_2O)
Flow (L/s)
Time (s)
