# Supplementary material for: Construction and Performance Testing of a Fast-Assembly COVID-19 (FALCON) Emergency Ventilator in a Model of Normal and Low-Pulmonary Compliance Conditions
Source: Front Physiol. 2021 Mar 22;12:642353. doi: 10.3389/fphys.2021.642353 (PMC8044930; doi:10.3389/fphys.2021.642353)
Supplement: Supplementary file 2 [file Data_Sheet_2.PDF]

Purchased parts list for constructing the FALCON prototype ventilator. Tools needed for assembly include diagonal pliers for cutting electrical wire, 1/8-inch flathead screwdriver for connecting electrical wire to the timer relay and 12-volt power supply, and Philips #2 screwdriver for connecting electrical wire to the PWMs. Additionally, 16-gauge electrical wire is used to connect the components. The prices listed are current as of January 10, 2021 and are subject to change.

Picture	Name	ID	Manufacturer	Price (USD)	Notes
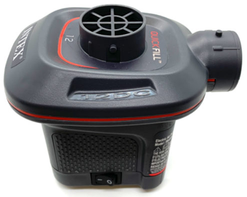	12-volt electric air pump	AP636	Intex®	11.99	—
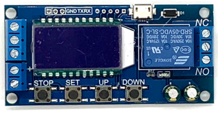	Timer relay	XY-LJ02	Belong International Co.	7.50	—
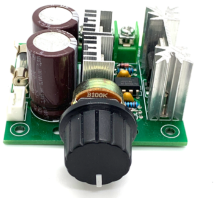	pulse width modulator	12-40V 10A PWM DC Motor Speed Controller with Knob	Riorand™	15.99 ea	Two are needed.
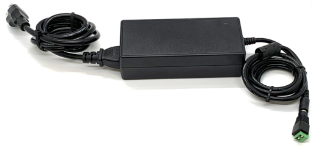	12-volt 8A power supply	SNT-1208	SHNITPWR Electronics Co.	18.99	—
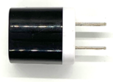	5-volt 1A USB power adapter	PT-WC-05	UorMe	1.98 ea	Available in a minimum pack of 6 (11.89 USD).
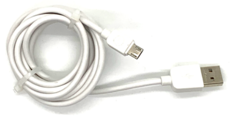	micro USB to USB A cable	HST-SMT3001	SMALLElectric	1.99 ea	Available in a minimum pack of 5 (9.99 USD).
