# Supplementary material for: Construction and Performance Testing of a Fast-Assembly COVID-19 (FALCON) Emergency Ventilator in a Model of Normal and Low-Pulmonary Compliance Conditions
Source: Front Physiol. 2021 Mar 22;12:642353. doi: 10.3389/fphys.2021.642353 (PMC8044930; doi:10.3389/fphys.2021.642353)
Supplement: Supplementary file 3 [file Data_Sheet_3.PDF]

# Detailed Assembly Instructions

# Step 1

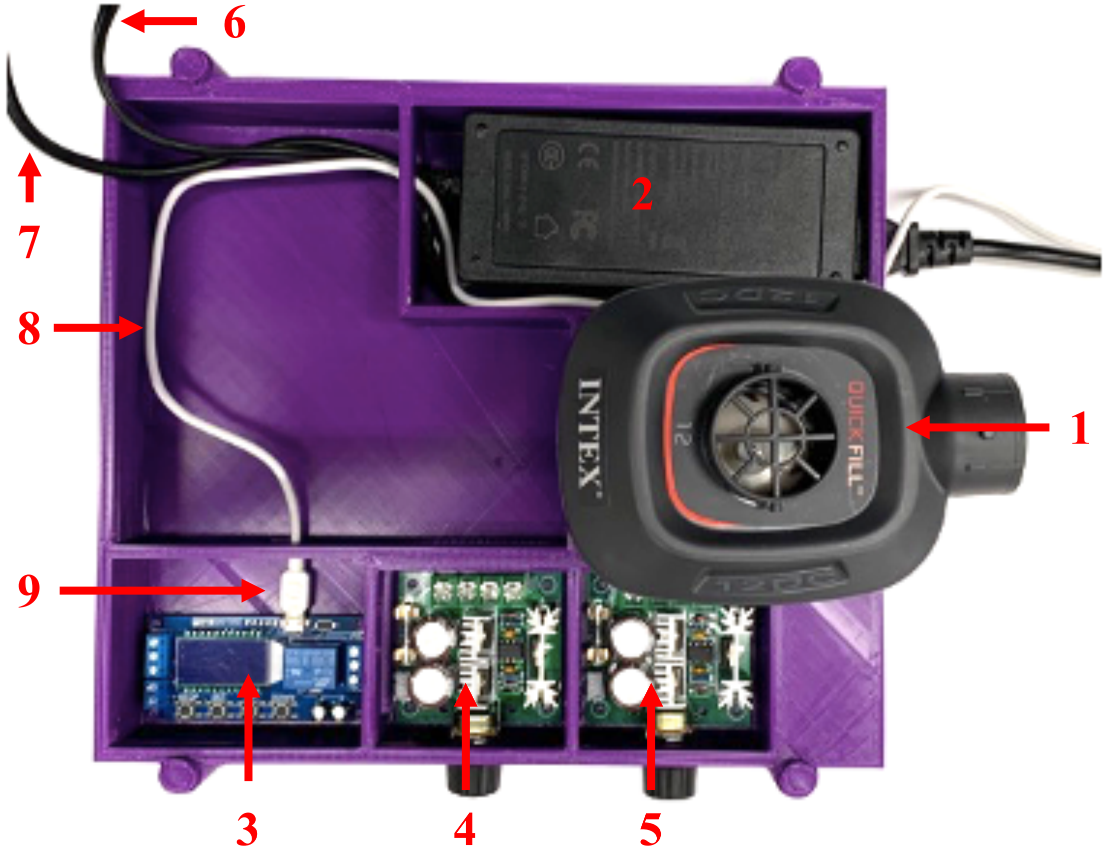


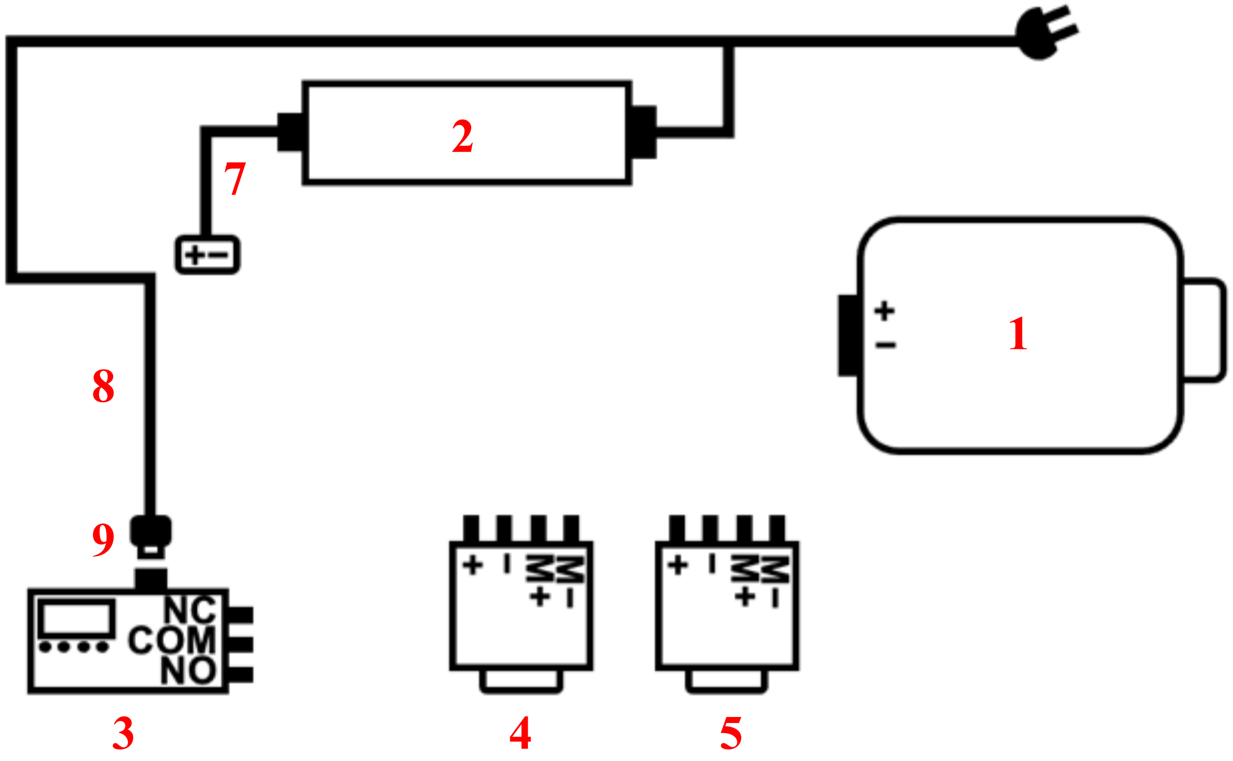


Place the electric air pump (1), 12V power supply (2), timer relay (3), and PIP (4) and PEEP (5) PWMs inside the 3D printed housing unit. Route the electric air pump cord (6, not shown in wire diagram) and the 12V (7) and mini USB 5V (8) power supply cords into the wire housing. Plug the mini USB 5V power supply into the timer relay (9). However, do not plug the power supplies into a power outlet at this time.

# Step 2

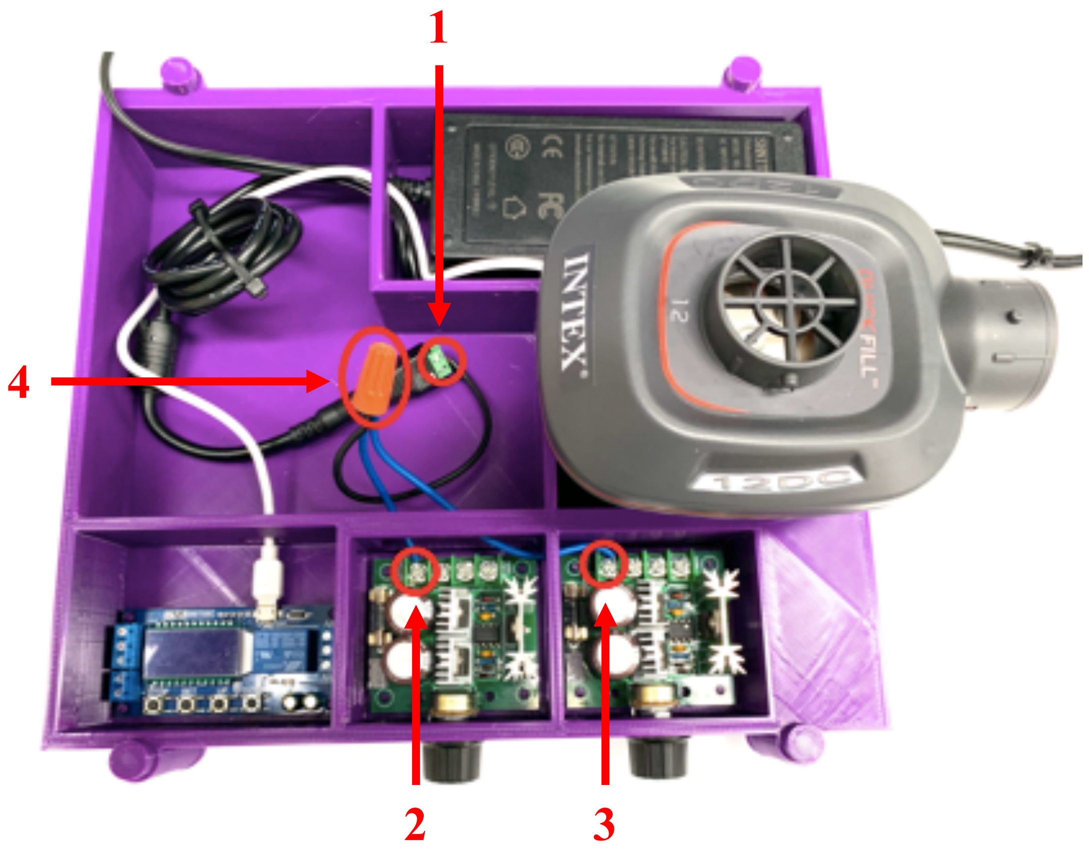


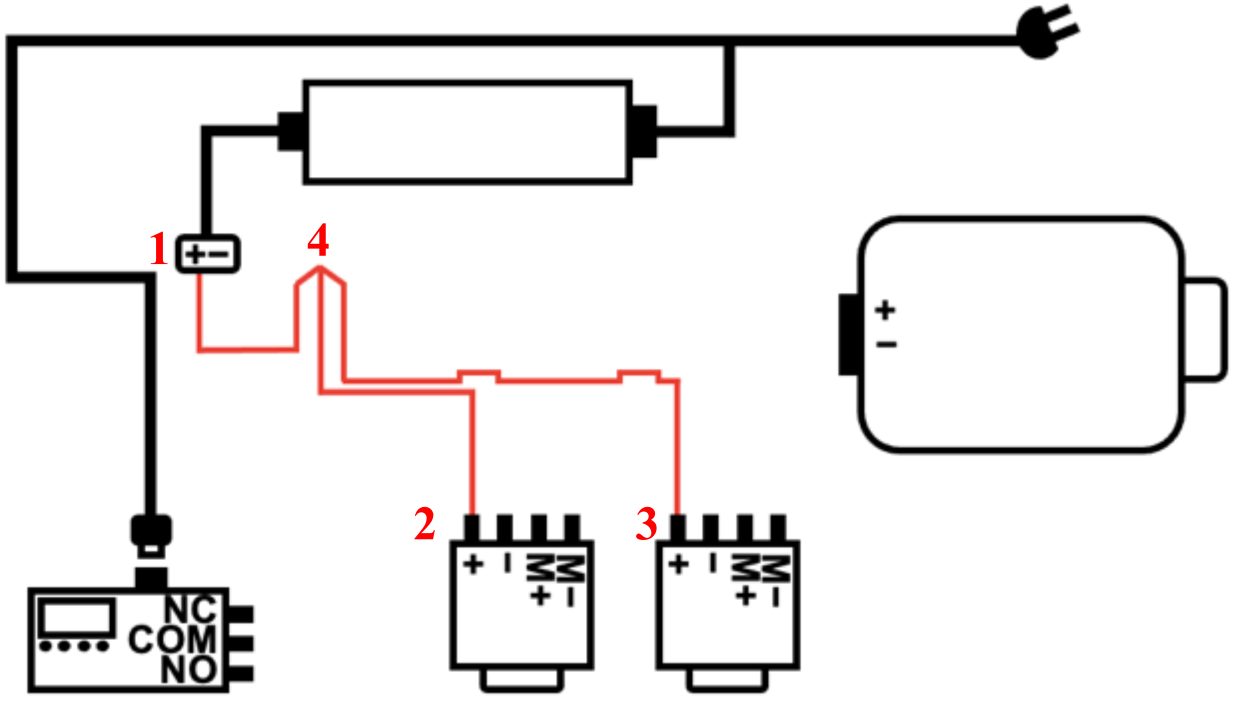


Connect the + end of the power supply (1) to the + terminals the PIP (2) and PEEP (3) PWMs using a screw-on wire cap connector (4).

# Step 3

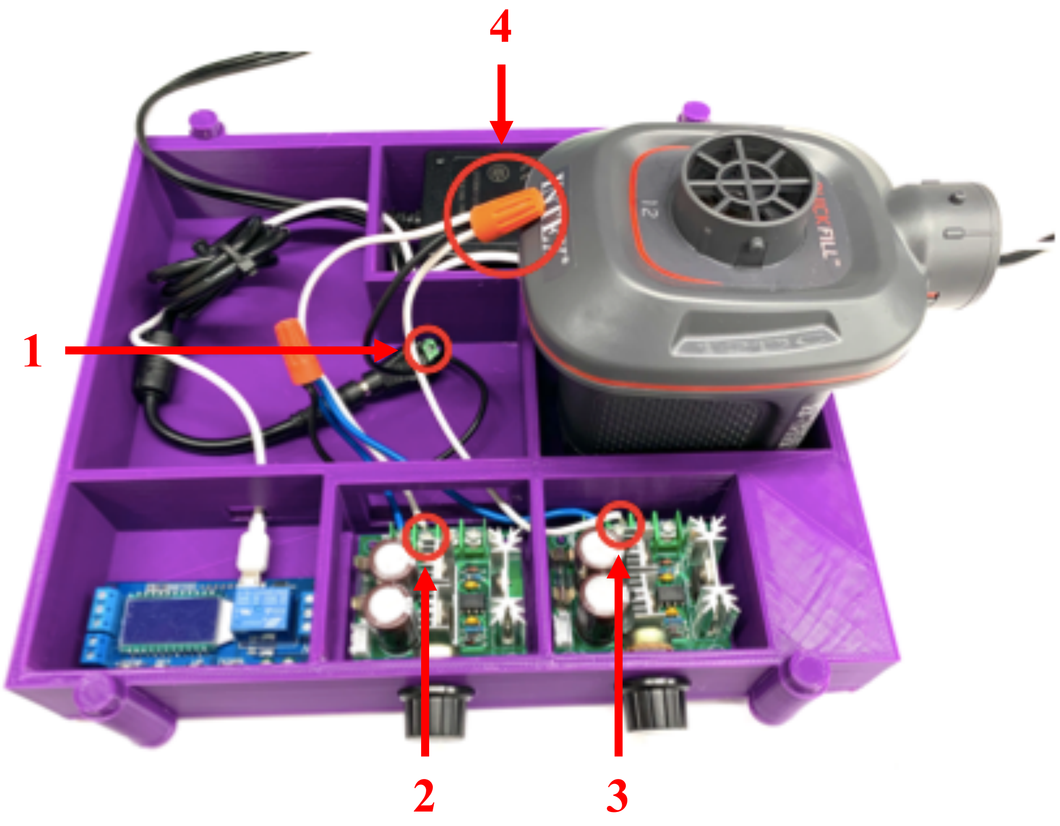


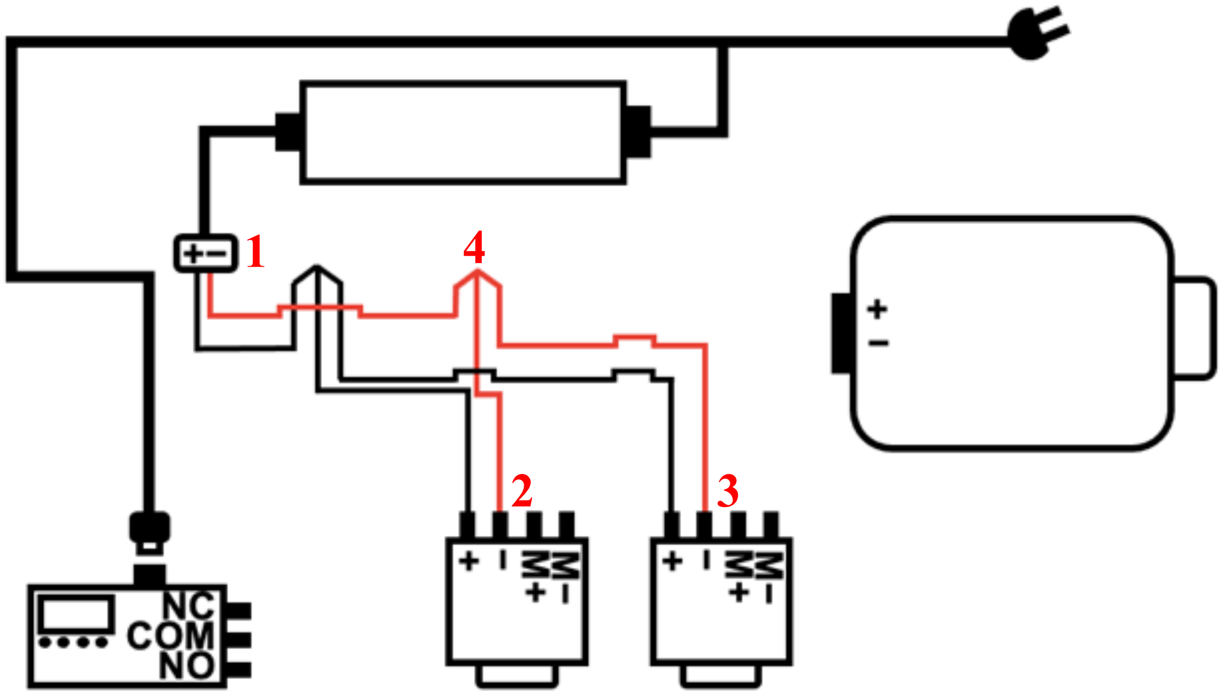


Connect the − end of the power supply (1) to the − terminals the PIP (2) and PEEP (3) PWMs using a screw-on wire cap connector (4).

# Step 4

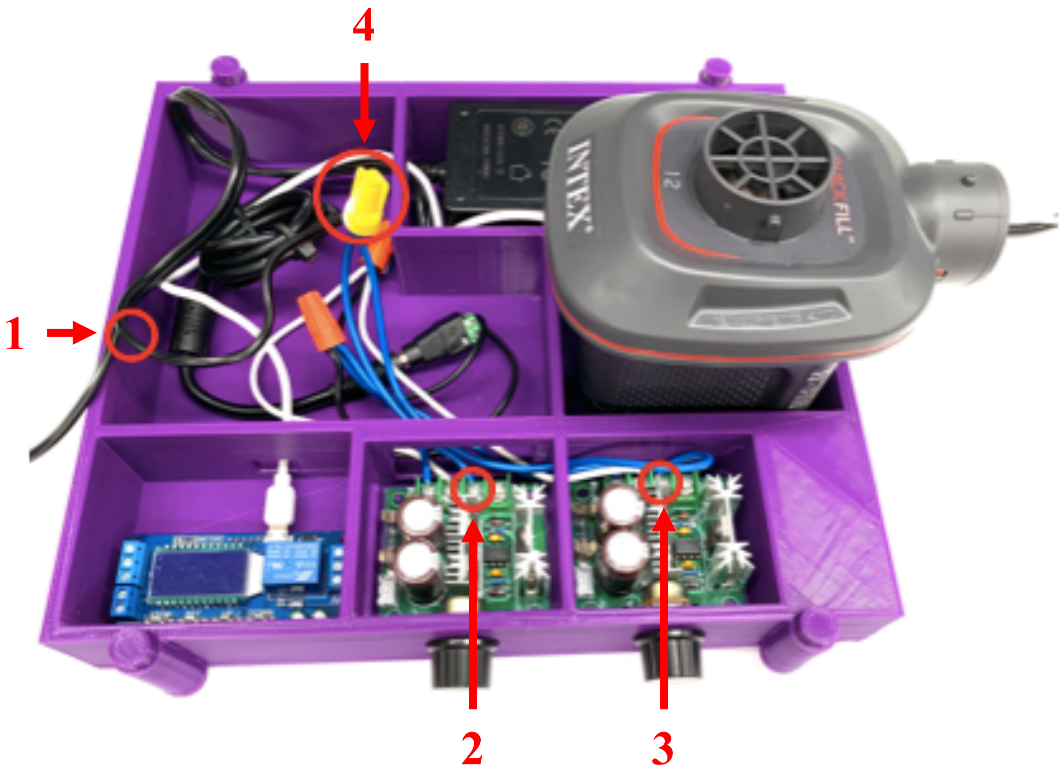


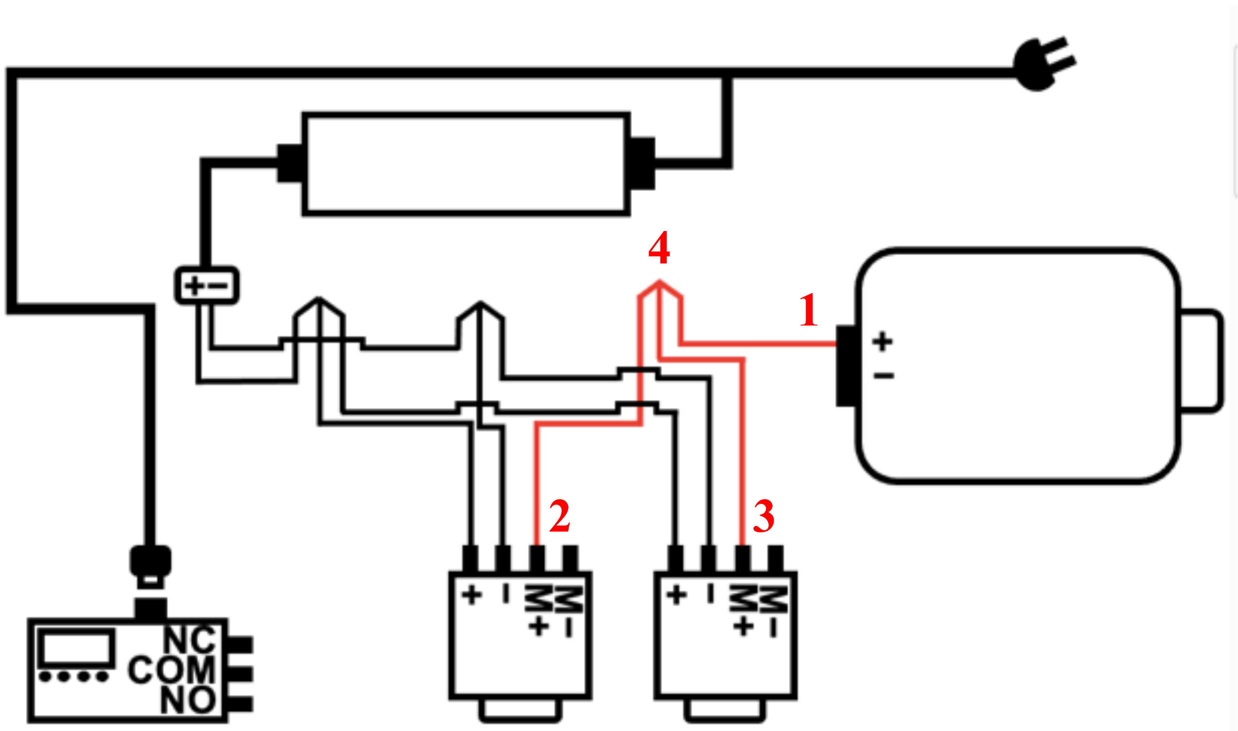


Connect the + end of the electric air pump (1) to the M+ terminals of the PIP (2) and PEEP (3) PWMs using a screw-on wire cap connector (4).

# Step 5

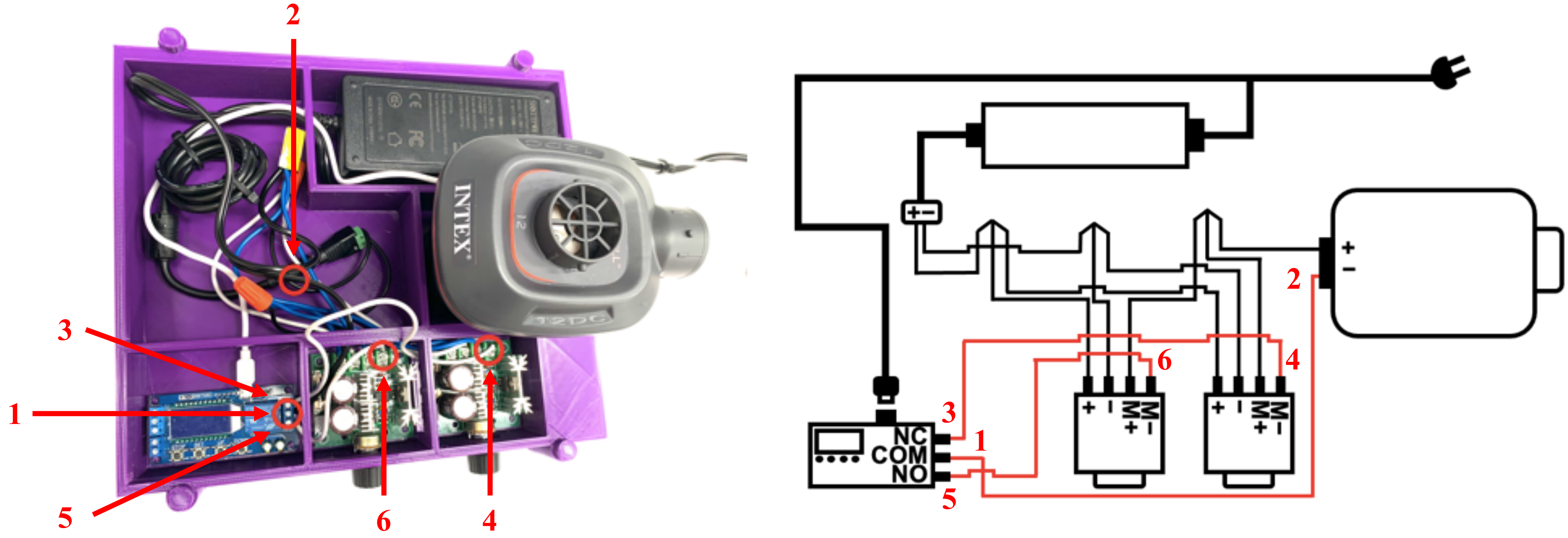


Connect the COM terminal of the timer relay (1) to the – end of the electric air pump (2). Connect the NC terminal of the timer relay (3) to the M– terminal of the PEEP PWM (4). Connect the NO terminal of the timer relay (5) to the M– terminal of the PIP PWM (6).
