# Supplementary material for: Construction and Performance Testing of a Fast-Assembly COVID-19 (FALCON) Emergency Ventilator in a Model of Normal and Low-Pulmonary Compliance Conditions
Source: Front Physiol. 2021 Mar 22;12:642353. doi: 10.3389/fphys.2021.642353 (PMC8044930; doi:10.3389/fphys.2021.642353)
Supplement: Supplementary file 4 [file Data_Sheet_4.PDF]

How to set the timer relay XY-LJ02

Plug the micro USB cable into the timer relay; the timer relay will remain powered indefinitely as long as the USB power adapter remains plugged into an electrical outlet.

Press and hold SET until “P1” starts blinking on the bottom left of the screen. Press UP/DOWN to reach “P6”. In this program, the “OPEN” state refers to the time during which the prototype delivers the set inspiratory pressure while the “CLOSED” state is the time during which the prototype delivers the set expiratory pressure.

Press SET again. Now the time, most likely “000.0”, should be blinking at the top and “OP” in the bottom right of the screen. To change the time in the “OPEN” state, press and hold UP/DOWN to the desired time. The “STOP” button can be pressed to change the precision of the set “OPEN” time. There are 4 possible precision settings that can be chosen:

1. “0000” there is no decimal point, so the highest precision is in seconds, e.g. “0198” is 198 seconds.

2. “000.0” the highest precision is in tenths of a second, e.g. “019.8” is 19.8 seconds. This was the setting that all bench tests were performed.

3. “00.00” the highest precision is in the hundredths of a second, e.g. “01.98” is 1.98 seconds.

4. “0.0.0.0” the highest precision is in minutes, e.g. “0.1.9.8” is 198 minutes.

Press SET to confirm the time in the “OPEN” state and to set the time in the “CLOSED” state. “CL” should now be blinking in the bottom right of the screen. Again, press and hold UP/DOWN to the desired time. The “STOP” button can be pressed to change the precision of the set “CLOSED” time.

Press SET once more to confirm the time in the “CLOSED” state. “LP” should now be blinking in the bottom right of the screen, and this refers to the number of loops that the program cycles between the “OPEN” and “CLOSED” state. To cycle between the two indefinitely, press and hold DOWN until “- - - -” appears on the screen.

Press and hold SET to save the settings. After releasing the SET button, the timer relay should immediately begin cycling between the “OPEN” and “CLOSED” states.
