# Supplementary material for: Construction and Performance Testing of a Fast-Assembly COVID-19 (FALCON) Emergency Ventilator in a Model of Normal and Low-Pulmonary Compliance Conditions
Source: Front Physiol. 2021 Mar 22;12:642353. doi: 10.3389/fphys.2021.642353 (PMC8044930; doi:10.3389/fphys.2021.642353)
Supplement: Supplementary file 5 [file Data_Sheet_5.PDF]

Failure Modes, Effects, and Controls Prospectus

Process	Process Step	Potential Failure Mode	Potential Effect(s) of Failure	Potential Cause(s) of Failure	Current Process Controls
Assembly	Assembly Step 1. Place the electric air pump, 12V power supply, timer relay, and PIP and PEEP PWMs inside the 3D printed housing unit. Route the electric air pump cord and the 12V and mini USB 5V power supply cords into the wire housing.	Defect in air pump.	Air pump will not turn on. Air pump cannot generate high enough pressures.	Defective air pump. Defective or worn wiring.	Test prior to use and replace defective component.
		Defect in 12V power supply.	No power to PWMs and air pump.	Defective power supply block. Defective or worn wiring.	
		Defect in power supply outlet cord.	No power to PWMs and air pump.	Defective or worn wiring.	
		Defect in timer relay.	Timer relay will not turn on. Ventilator will not switch from PEEP to PIP pressure.	Defective component.	
		Defect in PIP PWM.	Air pump will not turn on during inspiration. PIP cannot be predictably controlled.		
		Defect in PEEP PWM.	Air pump will not turn on during expiration. PEEP cannot be predictably controlled.		
	Assembly Step 1 (cont'd). Plug the mini USB 5V power supply into the timer relay.	Defect in 5V power supply.	Timer relay will not turn on.	Defective power adapter. Defective or worn USB power cord.	
	Assembly Step 2. Connect the + end of the power supply to the + terminal of the PIP PWM using a screw-on wire cap connector.	Connection from + end of power supply to PIP PWM not secure.	PIP not generated during inspiration. Air pump will stutter during insiration, leading to inconsistent PIP.	Wire connection at + end of power supply not securely connected. Wires at screw terminal not properly connected. Wire connection at + PIP PWM terminal not securely attached.	Pull on wires close to connection to ensure proper connection to terminals.

Process	Process Step	Potential Failure Mode	Potential Effect(s) of Failure	Potential Cause(s) of Failure	Current Process Controls
Assembly	Assembly Step 2 (cont’d). Connect the + end of the power supply to the + terminal of the PEEP PWM using a screw-on wire cap connector.	Connection from + end of power supply to PEEP PWM not secure.	PEEP not generated during inspiration. Air pump will stutter during expiration, leading to inconsistent PEEP.	Wire connection at + end of power supply not securely connected. Wires at screw terminal not properly connected. Wire connection at + PEEP PWM terminal not securely attached.	Pull on wires close to connection to ensure proper connection to terminals.
	Assembly Step 3. Connect the − end of the power supply to the – terminal of the PIP PWM using a screw-on wire cap connector.	Connection from − end of power supply to PIP PWM not secure.	PIP not generated during inspiration. Air pump will stutter during insiration, leading to inconsistent PIP.	Wire connection at − end of power supply not securely connected. Wires at screw terminal not properly connected. Wire connection at − PIP PWM terminal not securely attached.	
	Assembly Step 3 (cont’d). Connect the − end of the power supply to the – terminal of the PEEP PWM using a screw-on wire cap connector.	Connection from − end of power supply to PEEP PWM not secure.	PEEP not generated during inspiration. Air pump will stutter during expiration, leading to inconsistent PEEP.	Wire connection at − end of power supply not securely connected. Wires at screw terminal not properly connected. Wire connection at − PEEP PWM terminal not securely attached.	
	Assembly Step 4. Connect the + end of the electric air pump to the M+ terminal of the PIP PWM using a screw-on wire cap connector.	Connection from + end of air pump to PIP PWM M+ terminal not secure.	PIP not generated during inspiration. Air pump will stutter during insiration, leading to inconsistent PIP.	Wire connection at + end of air pump not securely connected. Wires at screw terminal not properly connected. Wire connection at M+ PIP PWM terminal not securely attached.	
	Assembly Step 4 (cont’d). Connect the + end of the electric air pump to the M+ terminal of the PEEP PWM using a screw-on wire cap connector.	Connection from + end of air pump to PEEP PWM M+ terminal not secure.	PEEP not generated during inspiration. Air pump will stutter during expiration, leading to inconsistent PEEP.	Wire connection at + end of air pump not securely connected. Wires at screw terminal not properly connected. Wire connection at M+ PEEP PWM terminal not securely attached.	

Process	Process Step	Potential Failure Mode	Potential Effect(s) of Failure	Potential Cause(s) of Failure	Current Process Controls
Assembly	Assembly Step 5. Connect the COM terminal of the timer relay to the – end of the electric air pump.	Connection from − end of air pump to timer relay COM terminal not secure.	Air pump will not turn on. Air pump will stutter, leading to inconsistent PIP and/or PEEP.	Wire connection at COM terminal not securely connected.	Pull on wires close to connection to ensure proper connection to terminals.
	Assembly Step 5 (cont'd). Connect the NC terminal of the timer relay to the M– terminal of the PEEP PWM.	Connection from timer relay NC terminal to M− terminal of PEEP PWM not secure.	PEEP not generated during inspiration. Air pump will stutter during expiration, leading to inconsistent PEEP.	Wire connection at timer relay NC terminal not securely attached. Wire connection at M− terminal of PEEP PWM not securely attached.	
	Assembly Step 5 (cont'd). Connect the NO terminal of the timer relay to the M– terminal of the PIP PWM.	Connection from timer relay NO terminal to M− terminal of PIP PWM not secure.	PIP not generated during inspiration. Air pump will stutter during expiration, leading to inconsistent PIP.	Wire connection at timer relay NO terminal not securely attached. Wire connection at M+ terminal of PIP PWM not securely attached.	
Setup	Setup Step 1. Place FALCON ventilator next to patient and breathing circuit.	Obstruction of air pump inlet.	Air pump cannot pull necessary air to properly function, leading to inadequte pressure generation.	Placement of ventilator next to object(s) that can obstruct air pump inlet.	Place ventilator away from objects that may obstruct air pump inlet.
	Setup Step 2. Plug 12V power supply into into wall outlet.	Wall outlet does not supply power to 12V power supply.	Ventilator air pump will not turn on or operate.	Building power outage. Wall outlet defective.	Test wall outlet prior to use. Ensure manual ventilation equipment or other ventilatory option is available for emergency ventilation if power outage occurs.
	Setup Step 3. Plug 5V power adapter into wall outlet	Wall outlet does not supply power to 5V power supply.	Ventilator timer relay will not turn on. Air pump will only generate constant PEEP pressure.		
	Setup Step 4. Attach breathing circuit to ventilator air pump outlet.	Breathing circuit is attached to ventilator air pump inlet.	Air will be pulled from breathing circuit rather than pushed, leading to ventilation failure.	Accidental attachment to the air pump inlet.	Test ventilator prior to patient connection to ensure air is being pushed into breathing circuit.

Failure Modes, Effects, and Controls Prospectus

Process	Process Step	Potential Failure Mode	Potential Effect(s) of Failure	Potential Cause(s) of Failure	Current Process Controls
Setup		Air leak between the air pump and the breathing circuit.	Inadequate pressure generation. Uncontrolled aerosolization of infective agents and exposure to nearby patients and healthcare personnel.	Inappropriate sizing of breathing circuit tubing. Tubing is not adequately attached to air pump outlet. Tubing contains holes or cuts.	Ideally, use corrugated tubing with rubber end pieces to minimize risk of leak. Otherwise, ensure properly sized ventilator tubing is used and inspect prior to use.
	Setup Step 5: Attach pressure gauge to breathing circuit.	Inaccurate pressure measurements.	Unintended pressures delivered to patient.	Imprecise and/or inaccurate pressure gauge. User misreads pressure gauge. Pressure gauge placed too far upstream of patient outlet, leading to a considerable pressure drop between the gauge and patient and lower-than-intended delivered pressures.	Use a well-calibrated pressure gauge and place in breathing circuit as close as feasibly possible to patient outlet.
	Setup Step 6. Set timer relay to correct mode.	Incorrect mode set in timer relay.	Timer relay will not cycle between inspiration and expiration, leading to no ventilation.	User unintentionally selects incorrect mode on the timer relay. User misunderstands timer relay instructions provided in the supplementary material.	Review supplementary instructions for setting timer relay parameters and test prior to patient hook up.
	Setup Step 7. Set timer relay inspiratory and expiratory times.	Incorrect inspiratory/expiratory time set in timer relay.	Inspiration/expiration time too long or short, leading to ineffecient ventilation.	User unintentionally selects inappropriate time on the timer relay. User misunderstands timer relay instructions provided in the supplementary material.	
		Timer relay set to inappropriate unit of time.	Inspiration/expiration time too long or short by order(s) of magnitude, leading to ineffective ventilation.	User unintentionally selects inappropriate unit of time (e.g. minutes instead of seconds). User misunderstands timer relay instructions provided in the supplementary material.	
	Setup Step 8. Set timer relay LOOP to infinite.	Timer relay LOOP not set to infinite.	Ventilator may eventually stop after the set number of loops have occurred.	User forgets to set LOOP to infinite. User misunderstands timer relay instructions provided in the supplementary material.	

**Process**	**Process Step**	**Potential Failure Mode**	**Potential Effect(s) of Failure**	**Potential Cause(s) of Failure**	**Current Process Controls**
Setup	Setup Step 9: Test ventilator settings prior to patient hook up.	Ventilator is not tested prior to patient hook up.	Ventilator settings are not set as intended, and ventilation may fail.	User forgets to test prior to patient hook up.	User must ensure that the ventilator is tested prior to patient hook up.
	Setup Step 10. Confirm timer relay settings to start ventilation.	Timer relay settings are not confirmed.	Ventilation does not start.	User forgets to press the button on the timer relay to save settings and begin ventilation. User misunderstands timer relay instructions provided in the supplementary material.	Review supplementary instructions for setting timer relay parameters and test prior to patient hook up.
	Setup Step 11. Attach patient to breathing circuit	Air leak between patient and breathing circuit.	Inadequate pressure generation. Uncontrolled aerosolization of infective agents and exposure to nearby patients, healthcare personnel.	Inappropriate sizing of breathing circuit tubing. Tubing is not adequately attached to patient tube. Tubing contains holes or cuts.	Ensure properly sized tubing is used and inspect prior to use.
		Air entering/leaving breathing circuit not appropriately filtered.	Uncontrolled aerosolization of infective agents and exposure to nearby patients and healthcare personnel.	Air filter(s) not used. Air filter(s) defective or inadequate.	Use unexpired respiratory HEPA filter(s) to minimize risk of aerosolization. Do not reuse filters that are meant for single use only.
During Ventilation	During Continuous Mandatory Ventilation.	12V power supply loss to ventilator.	No power to PWMs and air pump.	Building power outage. Wall outlet defective.	Test wall outlet prior to use. Ensure manual ventilation equipment is available for emergency ventilation if power outage occurs.
		Loss of 5V power supply.	Timer relay will not turn on or operate.		
		Loose wiring connection.	Effects of failure may be the same as described in the assembly failure.	Ventilator is dropped or connections lost. Additional causes of failure identical to the ones described in the assembly steps.	Minimize risk of accidental disconnection (for example, place ventilator away from high traffic area). During assembly, pull on wires close to connection to ensure proper connection to terminals.

Process	Process Step	Potential Failure Mode	Potential Effect(s) of Failure	Potential Cause(s) of Failure	Current Process Controls
During Ventilation	During Continuous Mandatory Ventilation.	Ventilator short circuits.	Electronic component(s) fail.	Water, blood, or other bodily fluids spill onto the ventilator. Ventilator is agitated, loosening wire connections, and leading to inappropriate wire contact.	Minimize risk of fluid exposure (for example, do not place containers of water near ventilator). If unit becomes soiled, switch to another ventilatory option (manual ventilation or another ventilator), unplug ventilator, and remove from service. Remove ventilator housing cover and inspect components for exposure. Clean and sanitize or simply replace non-electronic components. Replace soiled electronic components. Reassemble ventilator and test prior to reuse.
		Ventilator exposure to potentially infectious material (blood, respiratory secretions, other bodily fluids).	Ventilator fails. Ventilator becomes a vector for disease transfer to subsequent patients and personnel.	Exuberant respiratory secretion production, tracheostomy decannulation, chest compressions during cardiopulmonary resuscitation.	If unit becomes soiled, switch to another ventilatory option (manual ventilation or another ventilator), unplug ventilator, and remove from service. Remove ventilator housing cover and inspect components for exposure. Clean and sanitize or simply replace non-electronic components. Replace soiled components. Reassemble ventilator and test prior to reuse.
		Pressures drift away from originally set pressures.	Inappropriate PIP/PEEP delivered to patient.	Patient airway resistance has changed. PIP/PEEP PWMs have been unintentionally changed. PWMs and/or 12V power supply do not supply consistent current to air pump.	Periodically check pressure gauge for appropriate and consistent pressures during a respiratory cycle. Check for leaks in hosing and connections.

Failure Modes, Effects, and Controls Prospectus

Process	Process Step	Potential Failure Mode	Potential Effect(s) of Failure	Potential Cause(s) of Failure	Current Process Controls
During Ventilation	During Continuous Mandatory Ventilation.	Patient disconnected from breathing circuit	Patient apnea.	Breathing circuit is jostled. Breathing circuit not well-connected to patient. Lack of disconnect alarm.	Minimize risk of accidental jostling by patient and/or personnel.
		Insufficient tidal volume delivery.	Ineffective ventilation.	Lack of tidal volume monitoring requires periodic reassessment of PIP/PEEP settings.	Visually inspect patient for rise and fall of chest. If available, utilize a tidal volume measuring device.
		Patient too lightly sedated/paralyzed.	Patient/ventilator dyssynchrony.	Inadequate sedative(s)/paralytic(s) administered.	Observe patient for signs of patient/ventilator dyssynchrony (dyssynchronous patient breathing effort, apparent patient discomfort, unexpected fluctuations in pressures as observed on pressure gauge).
		PIP/PEEP inappropriately set.	Ineffective ventilation, ventilator-induced lung injury, pneumothorax	Ventilator or PWMs are jostled or damaged. User inappropriately sets PIP/PEEP for current clinical scenario.	Periodically check pressure gauge for appropriate and consistent pressures during respiratory cycling. Periodically check for signs of pneumothorax (decreased breath sounds on affected side, tachycardia, hypotension); diagnosis can be confirmed with chest X-ray.
		Inadequate FiO2.	Patient hypoxemia.	Progression of patient disease.	Monitor patient oxygen saturation using finger pulse oximetry, arterial blood gas measurement. If unable to manage, switch patient to a ventilator that can achieve an FiO2 greater than room air.
		Pressure required above max pressure of ventilator.	Inefficient ventilation.	Insufficient treatment of patient disease with progression.	Switch patient to a ventilator that can provide higher pressures.

Process	Process Step	Potential Failure Mode	Potential Effect(s) of Failure	Potential Cause(s) of Failure	Current Process Controls
During Ventilation	During Continuous Mandatory Ventilation.	Ventilator produces considerable electromagentic emissions.	Interference with proper functioning of other electronic-dependent medical equipment.	Ventilator untested for extent of electromagnetic emissions.	Place ventilator away from other electronic medical equipment. Test and shield ventilator against electromagnetic emissions prior to use.
		Ventilator affected by electromagnetic interference.	Interference with proper functioning of electrical components of ventilator, potentially leading to aberrant ventilator function and/or ventilator failure.	Ventilator is untested for electromagnetic interference.	Place ventilator away from other electronic medical equipment. Test ventilator for effects of electromagnetic interference prior to use.
		Air pump in ventilator not biocompatible.	Patient's airway exposed to emissions of outgassed volatile organic compounds (VOC).	Ventilator untested for biocompatibility and off-gassing of VOC.	Test ventilator for biocompatibility and release of VOC prior to use.
		Temperature and humidity variation.	Ventilator components could be adversely affected by extremes in temperature and humidity, potentially leading to aberrant ventilator function and/or ventilator failure.	Ventilator is untested for effects of extreme temperatures and humidity.	Test ventilator for effects of extremes in temperature and humidity prior to use.
	User changes timer relay settings during ventilation.	Settings on timer relay changed to too slow setting.	extended patient apnea.	User inputs incorrect information into timer relay and must first remedy change before restarting ventilation. User inexperienced in quickly inputting information into the timer relay.	Review supplementary instructions for setting timer relay parameters and practice changing settings prior to patient hook up.
		Settings on timer relay are inappropriately changed.	Inappropriate ventilation settings to patient. If LOOP setting or the timer relay mode is altered, ventilation may either immediately or eventually stop.	User incorrectly inputs information into timer relay.	

Failure Modes, Effects, and Controls Prospectus

**Process**	**Process Step**	**Potential Failure Mode**	**Potential Effect(s) of Failure**	**Potential Cause(s) of Failure**	**Current Process Controls**
During Ventilation	User changes PIP/PEEP during ventilation.	Inappropriate PIP/PEEP adjustment.	If corrected immediately, patients could be exposed to inappropriate PIP/PEEP for short periods of time. If uncorrected, this can lead to ineffective ventilation, ventilator-induced lung injury or pneumothorax.	User accidentally over-adjusts PIP/PEEP PWM settings. User unknowingly sets PIP/PEEP PWM(s) and does not check pressure gauge. User's target PIP/PEEP are inappropriate for patient.	Periodically evaluate pressure gauge to ensure ventilation is within appropriate and consistent pressure limits during respiratory cycling.
After Ventilation	Cleaning/Sterilization after use.	Ventilator ineffectively cleaned/sterilized.	Subsequent patients and personnel potentially exposed to infective agents.	Insufficient penetration of cleaning/sterilizing sprays/solutions.	Unplug ventilator. Remove and clean ventilator housing and breathing circuit as described in the manuscript. Remove ventilator housing cover and inspect components for contaminants. Manually clean and sanitize or replace soiled components. Reassemble ventilator and test prior to reuse.
